# Enzymatic and proteomic changes in resistant and susceptible cacao cultivars reveal distinct response mechanisms to *Phytophthora citrophthora* infection

**DOI:** 10.3389/fpls.2026.1718408

**Published:** 2026-03-09

**Authors:** Angra Paula Bomfim Rêgo, Irma Yuliana Mora-Ocampo, Elza Thaynara Cardoso de Menezes Assis, Márcia Christina da Silva Branco, Edna Dora Martins Newman Luz, Carlos Priminho Pirovani, Ronan Xavier Corrêa

**Affiliations:** 1 Centro de Biotecnologia e Genética, Universidade Estadual de Santa Cruz, Ilhéus, Brazil; 2 Centro de Pesquisas do Cacau, Comissão Executiva do Plano da Lavoura Cacaueira, Itabuna, Brazil; 3 Departamento de Ciências Biológicas, Universidade Estadual de Santa Cruz, Ilhéus, Brazil

**Keywords:** brown rot, gel-free proteomics, oxidative stress, plant disease, plant-pathogen interaction, resistance gene, sustainable agriculture, *Theobroma cacao*

## Abstract

Black pod rot, caused by *Phytophthora* species, is one of the most severe diseases affecting cocoa production. Among these species, *P. citrophthora* is considered one of the most aggressive, yet little is known about the molecular responses of cocoa to this pathogen. This study aimed to investigate the defense mechanisms of cacao against *P. citrophthora* through enzymatic analyses and gel-free comparative proteomics. Seedlings obtained by rooting cuttings from one resistant and one susceptible cultivar were inoculated with the pathogen, while controls received sterile distilled water. The activities of ascorbate peroxidase (APX), guaiacol peroxidase (GPX), catalase (CAT), and superoxide dismutase (SOD) were measured at 6, 12, 18, and 24 hours after inoculation (HAI). Protein abundance was evaluated at 24 HAI using mass spectrometry. The pathogen induced GPX activity from 6 HAI in the resistant and from 12 HAI in the susceptible cultivar, while APX activity increased in both cultivars after 18 HAI. A total of 1,583 proteins were identified across treatments. In the resistant cultivar, infection was associated with reduced photosynthesis, redirection of carbohydrate metabolism, and changes in the ascorbate/dehydroascorbate ratio, suggesting an efficient activation of defense responses. Constitutively abundant proteins related to antioxidant activity may also have contributed to resistance. In contrast, the susceptible cultivar showed limited protein abundance changes, with indications of increased metabolism of small molecules and accumulation of methylglyoxal, a cytotoxic compound linked to disease susceptibility. Overall, the results demonstrate that the resistant cultivar mobilizes early antioxidant defenses and metabolic reprogramming to cope with infection, whereas the susceptible exhibits inefficient responses leading to cellular damage. These findings provide new insights into cacao-*P. citrophthora* interactions, offer a foundation for future transcription-level studies, and may support the development of new pre-breeding stages for cacao cultivars.

## 1 Introduction

The global chocolate and confectionery market, valued at $100 billion, relies on about five million tons of cocoa beans (*Theobroma cacao* L.) produced annually (Winters et al., 2024). However, estimated losses of 30% to 90% of this production are caused by diseases from eight main species of the *Phytophthora* genus (Adeniyi, 2019; Decloquement et al., 2022; Perrine-Walker, 2020). Among these, *Phytophthora palmivora* (E.J. Butler) E.J. Butler 1919, *P. megakarya* Brasier & M.J. Griffin 1979, and *P. citrophthora* R.E.Sm & E.H. Sm. Leonian 1906 cause most losses in cocoa plantations in producing countries (McMahon and Purwantara, 2004; Marelli et al., 2019).

Among the oomycetes, the *Phytophthora* is notorious for causing devastating diseases in critical crops. It primarily affects the fruits in cocoa trees, leading to Black pod rot. The infection begins with small, hard, dark lesions that quickly expand, covering the entire fruit surface within a few days (Guest, 2007). The stem, flower cushions, leaves, and roots are less frequently affected (Surujdeo-Maharaj et al., 2016).

Molecular studies on cacao infected with *Phytophthora* spp. have revealed that the expression patterns of recognition receptors (PRRs) and a possible gene-for-gene interaction contribute to the cacao’s resistance to *Phytophthora* spp. Phenolic compounds play a crucial role in preformed defenses, and proline accumulation might be involved in maintaining cell wall integrity during infection (Rêgo et al., 2023). Studies on gene expression in the cocoa-*P. palmivora* interaction have revealed a dependence on the population origin of cocoa genotypes, and consequently, on the genetic and co-evolutionary processes involved in this pathosystem (Winters et al., 2024). Additionally, these studies reveal genes with constitutive expression patterns that differ between genotypes, as well as those induced by *P. palmivora* (Baruah et al., 2022).

Among the *Phytophthora* species affecting cacao trees, *P. citrophthora* is considered one of the most aggressive (Appiah et al., 2004). It was first described in California (Smith and Smith, 1906) when a new disease in citrus was reported (Mchau and Coffey, 1994). However, isolates previously identified as *P. citrophthora* infecting cacao have been recently reclassified through molecular identification as a new species: *P. theobromicola* sp. nov (Decloquement et al., 2022). Four distinctive characteristics were noted in this species infecting cacao: (i) only *P. citrophthora* isolates from cacao in Brazil produced chlamydospores; (ii) isolates from other hosts, including cacao from Indonesia, did not produce chlamydospores (Luz and Mitchell, 1994); (iii) isoenzyme variability analysis separated the electrophoretic types into three subgroups, one of which exclusively corresponded to *P. citrophthora* isolates from cacao in Brazil (Mchau and Coffey, 1994); (iv) only one study reported molecular responses of cacao to infection by *P. citrophthora*, identifying quantitative trait loci (QTL) corresponding to resistance to *P. citrophthora* (Barreto et al., 2018).

Understanding plant-pathogen interactions requires complementary molecular approaches. While transcriptomic analyses reveal which genes are expressed under specific conditions, proteomic profiling identifies which proteins are present and in what abundance, thereby clarifying their immediate functional contributions to cellular responses under contrasting conditions, incorporating the effects of post-translational regulation and protein-protein interactions. Additionally, through enzymatic activity analysis, various antioxidant enzymes can be used to examine the effects of biotic stresses. The study of plant-pathogen interactions at the proteomic level has uncovered valuable insights into crop improvement and the biotechnological potential of various proteins. Proteomic studies aimed at discovering the responses of cacao trees to infections by economically significant pathogens, such as *P. palmivora* (Rego et al., 2022), *Ceratocystis cacaofunesta* (Mora-Ocampo, 2020), and *Moniliophthora perniciosa* (Santos et al., 2020), have revealed different defense strategies employed by cacao trees for each pathogen. Therefore, it is crucial to understand the protein and enzymatic patterns in cacao’s interactions with its multiple pathogens. In the present study, the main objective was to evaluate the responses of cacao cultivars to infection by *P. citrophthora* through enzymatic activity and gel-free proteomics. We focus on elucidating the roles of various proteins involved in cacao resistance to this pathogen and propose possible mechanisms underlying this resistance.

## 2 Materials and methods

### 2.1 Plant material and cultivation conditions

The clonal cultivars of *T. cacao* used in this study exhibit contrasting resistance to *P. citrophthora*: the PH 16 cultivar is resistant (hereafter, R-PH 16), while the SJ 02 is susceptible (hereafter, S-SJ 16) (Lopez et al., 2006). In addition to their contrasting resistance, these two cultivars were chosen because they are widely used by many farmers in the cacao-producing regions of Southern Bahia. The cocoa seedlings were purchased from “Biofábrica da Bahia” (14°38’35”S 39°15’17”W), and the study was conducted at the Center for Biotechnology and Genetics (UESC, Ilhéus, Brazil; 14.7980° S, 39.1764° W) from March 2022 to February 2024, and was registered in SisGen (Register n. AAE2FED) for access to Brazilian genetic heritage.

Cuttings from plagiotropic branches of the two clonal cultivars were rooted in a substrate supplemented with coconut fiber and vermiculite and placed in 280 mL tubes, following standard seedling production procedures (Sodré and Marrocos, 2009). After six months, the seedlings were transplanted into 1-liter capacity bags containing a 1:1 mixture of autoclaved soil and substrate enriched with mineral macronutrients. The seedlings were then housed and grown in a greenhouse at the State University of Santa Cruz (UESC) in Ilhéus, BA, Brazil, with monitored temperatures (25-27 °C). Throughout the experimental period, the seedlings were watered once daily and fertilized with mineral macronutrients every 15 days until the experiment concluded. Fifteen-month-old seedlings, visually uniform in size and exhibiting at least five leaves with dark green leaf blades, were selected for the inoculation treatments. We inoculated the leaves directly onto the seedling. Since these are seedlings propagated from cuttings of the same mother plant of each cultivar, they are genetically identical. To control age, we included seedlings that produced simultaneous vegetative shoots in the experiment. Therefore, all the leaves used in the experiment were the same age.

### 2.2 Acquisition of inoculum, inoculation, and collection of plant material

The *P. citrophthora* isolate number 1893 was obtained from the *Phytophthora* Collection of the Plant Pathology section at CEPEC/CEPLAC. The methodologies for the isolate’s viability and pathogenicity tests, production of the zoospore suspension (3 x 10^5^ zoospores mL-1 of sterile water), and seedling inoculation were developed by Luz et al. (2008).

The inoculations were carried out by manually spraying the leaves of seedlings around 15 months old. Forty-eight seedlings of each cultivar (PH 16 and SJ 02) were inoculated with the pathogen, and another 48 seedlings of each cultivar were inoculated with sterile distilled water as a control treatment.

Leaf samples for molecular analyses were collected at four time points: 6, 12, 18, and 24 hours after inoculation (HAI) with *P. citrophthora*. At each point, two leaves were harvested from each of 12 independent biological replicates per genotype, under both experimental conditions: (i) inoculation with *P. citrophthora* zoospores and (ii) inoculation with sterile distilled water (control). The collected leaf samples were immediately frozen in liquid nitrogen, lyophilized, and stored at -80 °C until preparation for enzymatic and molecular analyses. This sampling strategy resulted in a total of 24 leaves per treatment and genotype at each collection time, which were subsequently pooled for downstream analyses.

### 2.3 Enzyme activity

Enzyme extracts were obtained by macerating leaf samples in liquid nitrogen using a mortar and pestle. Subsequently, forty milligrams of each sample were weighed on an analytical balance, and polyvinylpolypyrrolidone (PVPP) at a ratio of 0.7g/g of leaf tissue was added to the sample. The samples were homogenized in 0.8 mL of specific buffer solution for each enzyme: potassium phosphate (50 mM, pH 7.8) for superoxide dismutase (SOD, EC 1.15.1.1); potassium phosphate (50 mM, pH 7.0) for ascorbate peroxidase (APX, EC 1.11.1.11) and catalase (CAT, EC 1.11.1.6); and sodium phosphate (50 mM, pH 6.0) for guaiacol peroxidase (GPX, EC 1.11.1.7). Next, the samples were resuspended by sonication (8 pulses of 5 seconds each, 70% output, with 10-second intervals) using an ultrasonic processor (Gex 130) and centrifuged at 10,000 x g for 10 minutes at 4°C. The supernatant (crude extract) was transferred to a new tube and kept on ice until analysis. The activities of SOD, APX, CAT, and GPX were evaluated. SOD activity was measured according to Giannopolitis and Ries (1977); APX activity was determined following Nakano and Asada (1987); CAT activity was assessed using the method described by Havir and McHale (1987), and GPX activity was evaluated according to the protocol by Rehem et al. (2012). For each enzyme, polystyrene microplates were prepared with each well containing crude extract and the necessary reaction mix to initiate enzymatic activity, with three technical replicates per method. Plate readings were performed using a UV/Vis spectrophotometer (Spectramax Paradigm Multi-Mode Microplate Detection Platform, Molecular Devices). One unit of SOD activity (UA) was defined as the amount of sample required to inhibit 50% of the NBT photoreduction to blue formazan, and the results were expressed in UA g^-1^ fresh weight. APX activity was calculated using the molar extinction coefficient of 2.8 mmol^-1^ L cm^-1^ and expressed in mmol ascorbate min^-1^ g^-1^ fresh weight. CAT activity was expressed in mmol H_2_O_2_ min^-1^ g^-1^ fresh weight, using the molar extinction coefficient of 36 M^-1^ cm^-1^. For GPX, the conversion of absorbance data at 470 nm min^-1^ g^-1^ fresh weight to guaiacol consumption in mmol min^-1^ g^-1^ fresh weight was made using the equation y=0.8382.ABS + 0.1324 (R^2^ = 0.99).

#### 2.3.1 Experimental design and statistical testing

A completely randomized design was employed in a 2 x 2 x 4 factorial arrangement for the statistical analysis of enzyme activities. This involved two cultivars (PH 16 and SJ 02), two treatments (control and inoculated), and four time points (6, 12, 18, and 24 HAI), resulting in 16 treatment combinations with 48 replications per experimental unit. The experimental results were subjected to analysis of variance (ANOVA). Multiple mean comparisons were performed using Tukey’s test (p<0.05).

#### 2.3.2 Biplot analysis based on principal component analysis

To evaluate the interrelationships between treatments and biochemical variables (APX, GPX, CAT, and SOD), a biplot analysis based on Principal Component Analysis (PCA) was conducted using Statistica 7.0 software. Initially, the values of each variable were standardized to a mean of 0 and a variance of 1 using the equation Z_ij_= (X_ij_ – μ_j_)/S_j_, where X_ij_ is the value of the i-th observation of variable X_j_, and μ_j_ and S_j_ are the mean and standard deviation of Xj. Subsequently, the standardized values were subjected to Multivariate Factor Analysis using factor loadings > 0.70 as the criterion. Among the variables initially analyzed, only APX, GPX, and SOD showed factor loadings > 0.70. Thus, these variables were selected for the Principal Component Analysis (active variables). The variable not selected by Factor Analysis (CAT) was used as a supplementary variable. Although the CAT variable was not used to extract the principal components, its relationship with the various treatments can be evaluated simultaneously with the others. For each variable (active or supplementary), the mean of each treatment was used as input data in the PCA, and the values were obtained from the singular value decomposition.

### 2.4 Gel-free proteomics

#### 2.4.1 Protein extraction

Based on the biplot analysis from PCA, the 24-hour collection time was chosen for proteomic analysis. Total protein extraction from the samples followed the protocol developed by Pirovani et al. (2008). To prevent oxidation, 0.2 g of the lyophilized sample was ground with liquid nitrogen and polyvinylpolypyrrolidone (PVPP). Subsequently, the samples were washed to remove pigments and other interfering substances, then homogenized by sonication and precipitated. Proteins were extracted using SDS extraction buffer and phenol and then precipitated with 0.1 mol L^-1^ ammonium acetate in methanol. The protein precipitates were recovered by centrifugation, washed, and resuspended in 400 μL of 8 mol L^-1^ urea. Protein quantification was performed using the 2D Quant Kit (Cytiva) according to the manufacturer’s instructions.

#### 2.4.2 SDS-PAGE gel electrophoresis

Following the quantification step, 40 μg of protein from each sample was analyzed using SDS-PAGE (Sodium Dodecyl Sulfate - Polyacrylamide Gel Electrophoresis) in mini electrophoresis tanks (Omniphor), with 8 x 10 cm gels containing 12.5% acrylamide as per Laemmli (1970). The gels were stained with 0.08% colloidal Coomassie, according to Neuhoff et al. (1988).

#### 2.4.3 Peptide digestion

An 80-µg protein mass from each sample was used for trypsin digestion, carried out according to the methodology described by Villén and Gygi (2008) with modifications. Briefly, the samples were diluted in water at a 1:1 ratio, followed by protein reduction with dithiothreitol (DTT) and alkylated with iodoacetamide (IAA). The samples were then diluted at a 1:5 ratio with 50 mmol L^-1^ ammonium bicarbonate and - calcium chloride (CaCl2) was added. Subsequently, they were incubated with trypsin at 37 °C for 16–24 hours. The reaction was stopped by adding trifluoroacetic acid (TFA) until the pH dropped below 2.0. The solution containing the tryptic peptides was desalted using C18 resin tips (100 µL; Thermo Fisher^®^), following the manufacturer’s recommendations. The peptides were eluted in 50 µL of a solution containing 50% acetonitrile in water with 0.1% formic acid.

#### 2.4.4 Mass spectrometry

The peptides were analyzed using a liquid chromatography system (Agilent 1290 Infinity II HPLC) coupled with a quadrupole/Time-of-Flight mass spectrometer (Agilent 6545 LC/QTOF). Eight microliters of each sample were injected in technical triplicates. The peptides were separated on a reverse-phase column (C18; AdvanceBio Peptide Mapping 2.1 x 250 mm; Agilent) maintained at a temperature of 55 °C. A 20-minute gradient was applied with mobile phases A (H2O with 0.1% formic acid) and B (acetonitrile with 0.1% formic acid). The phase B percentages over the gradient were 5% to 35% (1–10 min), 35% to 70% (11–14 min), 70% to 100% (16–18 min), and 100% (16–20 min). A final 5-minute period with 5% phase B was maintained to stabilize the pressure.

The samples were injected into the QTOF through an electrospray ionization source using the AutoMSMS acquisition mode, with a maximum of 10 precursors selected per cycle. The criteria for selecting precursors were: a threshold of 1000, 10,000 counts/spectrum, 100% purity stringency, 30% purity cut-off, isotopic model peptides, and charge preferences of 2, 3, >3, and unknown. The collision energy (in V) was set according to the formula:

(slope)*(m/z)/100+offset, where m/z represents the mass-to-charge ratio of the precursor, and the slope and offset vary from 3.1 to 5 and from -4.8 to 10, respectively, depending on whether the precursor charge is 2, 3, >3, or unknown. The instrument parameters used were gas temperature of 325 °C, gas flow of 13 L/min, capillary voltage of 4,000 V, and skimmer voltage of 56 V. Nitrogen gas was used for collision-induced dissociation. Instrument control (HPLC and QTOF) and the parameter settings were managed through the Agilent MassHunter Acquisition software.

### 2.5 Peptide identification in protein databases

The resulting spectra were processed in triplicate for peptide identification using the Spectrum Mill software from the Broad Institute (Rev BI.07.08.214). The parameters for spectrum extraction were: MSNoiseThreshold of 10 counts, fixed modifications of carbamidomethylation, precursor MH+ range of 200 to 6000 Da, retention time tolerance of +/- 60 seconds, m/z tolerance of +/- 1.4, and precursor charge state: find. After MS/MS spectra extraction, a database search was conducted. The database used was from *T. cacao*, downloaded from UniProt (https://www.uniprot.org) in February 2023. The parameters for MS/MS spectrum comparison in the protein database were: maximum number of missed cleavages: 2; fixed modifications: carbamidomethylation (C); variable modifications: oxidized methionine (M), pyroglutamic acid (N-termQ), deamidated (N), phosphorylated serine (S), phosphorylated threonine (T), phosphorylated tyrosine (Y); minimum combined peak intensity: 10%; precursor mass tolerance: +/- 20 ppm; product mass tolerance: +/- 50 ppm. The Spectrum-peptide matches (PSMs) from the search were validated and filtered using the Spectrum Mill’s Autovalidation tool for those with a false discovery rate (FDR) of less than 1%. For subsequent comparative statistical analysis, proteins containing peptides with a score > 5 and Scored Peak Intensity (SPI) >60% were selected. The proteins that passed these filters were exported in protein-protein comparison mode in MPP APR file format for further comparative analysis.

### 2.6 Identification of unique and differentially abundant proteins

Statistical analyses to identify differentially abundant proteins were conducted using Mass Profiler Professional 15.1 (MPP; Agilent). Protein abundance was calculated based on the median abundance of the peptides identified for each protein. This involved analyzing the valid proteins, filtered by score and SPI (item 2.4.4), across triplicate treatments (inoculated) and their respective control triplicates for each cultivar. A frequency filter was applied: only proteins present in at least two of the three technical replicates were considered for the unique entity analysis and statistical significance evaluation.

Statistical significance analyses for comparisons between treatment and control for each cultivar, as well as comparisons between controls of each one, were performed using an unpaired T-test. The p-value was calculated asymptotically, and multiple testing correction was done using the Benjamini-Hochberg method. Only common proteins between treatment and control for each cultivar, and between controls of each one, with a *p*-value< 0.05 and a fold-change ≥ 1.5 were considered differentially abundant.

For the clustering analysis, we considered the unique proteins after the frequency filter and the common proteins that were differentially abundant according to the aforementioned parameters. The parameters used for the clustering analysis were the normalized intensity values, Euclidean distance metric, and Ward’s linkage method.

### 2.7 Functional annotation and enrichment

The functional annotation of unique and differentially abundant proteins between the compared treatments was conducted using the free software ShinyGO v0.77 (Ge et al., 2019). Proteins not annotated using this software were searched on the UniProt website (https://www.uniprot.org/, accessed on February 19, 2023). Over-representation analysis of biological process categories, molecular function, and cellular components was performed using the BinGO tool in the Cytoscape software, applying the hypergeometric test with Benjamini & Hochberg correction (Maere et al., 2015).

### 2.8 Protein-protein interaction network

The protein-protein interaction network was constructed according to Mora-Ocampo et al. (2022) using the orthologs in Arabidopsis thaliana of the thioredoxin-dependent peroxiredoxin protein (A0A061EVI6) and cytochrome b6 (E3VU17). These proteins were identified as more abundant in the control treatment of the R-PH 16 than in the control of the S-SJ 02. They did not change significantly in the inoculated treatment of the R-PH 16. The String database (https://www.string-db.org/v.12.0, accessed on July 28, 2023) was used to obtain the interactions. Clustering and centrality parameters were calculated using the igraph package (Nepusz and Csárdi, 2006) in R software v.4.2.3 (R Core Team, 2022, and the network was then visually customized in Cytoscape software v.3.10.0 (Shannon, 2003). Functional enrichment analysis was performed using the BiNGO tool (Maere et al., 2015).

## 3 Results

### 3.1 Enzyme activity and biplot analysis based on principal component analysis

To evaluate the isolated and combined effects of independent variables (sources of variation) (**Table 1**) on the dependent variables of enzyme activity, the analysis of variance showed that APX activity was significantly influenced by the isolated effect of the cultivar and the interaction of C x T x HAI. In contrast, GPX and SOD activities were significantly influenced by the isolated effects of the cultivar, inoculation treatment, and time, as well as by the interaction among these factors. For CAT activity, no isolated effect of the cultivar was observed; only the effects of the treatment and time were significant. In this case, the effect of the cultivar on CAT activity was observed only in the interaction of C x HAI, which was the only significant interaction among the factors.

**Table 1:** ANOVA probability values for comparing the effects of cultivar (C), inoculation treatment (T), hours after inoculation (HAI), and the interactions C x T, G x HAI, T x HAI, and C x T x HAI on the activity of the enzymes ascorbate peroxidase (APX), guaiacol peroxidase (GPX), catalase (CAT), and superoxide dismutase (SOD) in cocoa cultivars (SJ 02 – susceptible – and PH 16 – resistant) inoculated with *P. citrophthora*.

Source of variation	APX	GPX	CAT	SOD
Cultivar (C)	0.0001^(^***^)^	0.0000^(^***^)^	0.3106^(ns)^	0.0000^(^***^)^
Treatment(T)	0.2265^(ns)^	0.0000^(^***^)^	0.0375^(^*^)^	0.0225^(^*^)^
Time (HAI)	0.0535^(ns)^	0.0000^(^***^)^	0.0011^(^**^)^	0.0000^(^***^)^
C x T	0.7774^(ns)^	0.0000^(^***^)^	0.1993^(ns)^	0.1919^(ns)^
C x HAI	0.0000^(^***^)^	0.0000^(^***^)^	0.0000^(^***^)^	0.0051^(^**^)^
T x HAI	0.0053^(^**^)^	0.0000^(^***^)^	0.5855^(ns)^	0.0000^(^***^)^
C x T x HAI	0.0008^(^***^)^	0.0000^(^***^)^	0.3891^(ns)^	0.0000^(^***^)^

(*) p<0.05; (**) p<0.01; (***) p<0.001; (ns) not significant.

**Figure 1:**
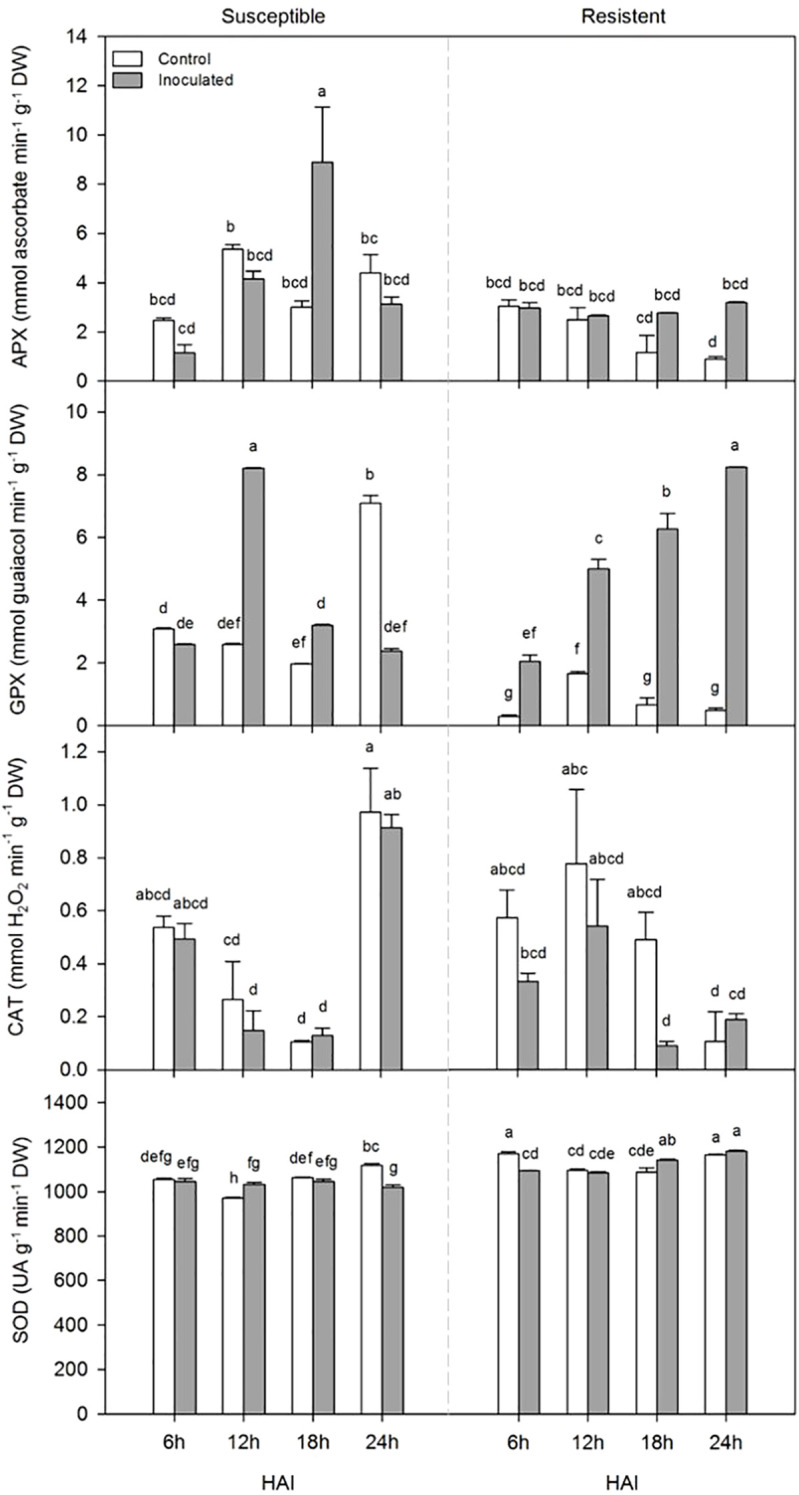
Activity of enzymes related to oxidative stress in *Theobroma cacao* plants. Activities of the enzymes ascorbate peroxidase (APX), guaiacol peroxidase (GPX), catalase (CAT), and superoxide dismutase (SOD) under different treatments: susceptible cultivar (SJ 02) inoculated with *P. citrophthora* and its control, and resistant cultivar (PH 16) inoculated with *P. citrophthora* and its control. Means followed by the same letters do not differ significantly according to Tukey’s test at a 5% probability level (p<0.05).

The activities of the enzymes APX, CAT, GPX, and SOD in cocoa showed significant differences across the variables analyzed for each cultivar (**Figure 1**). The activity of the enzyme APX was significantly influenced by the isolated effect of the cultivar and the interaction C x T x HAI. Within the cultivars, APX activity peaked at 18 h in the S-SJ 02 under the inoculated treatment, with the lowest enzyme activity at 6 h in the same treatment. At 24 h, S-SJ 02 increased its activity compared to the inoculated one at this time. The R-PH 16 inoculated had higher activity at 6h, decreased at 18h, and a trend towards recovery at 24h. According to Tukey’s test, the means for the inoculated treatment in PH 16 were similar (bcd), along with the controls at 6 h and 12 h. Only the control treatments at 18h and 24h were different. Resistant control treatment showed a gradual decrease in APX activity over time. In the evaluation of GPX activity, significant differences were observed when the cultivar, treatment, and collection time variables were evaluated together. Within the cultivars, higher GPX activity was observed in the inoculated treatments of both cultivars. Within each cultivar, the susceptible variety showed a peak in enzyme activity at 12h for the inoculated treatment, and the R-PH 16 showed a peak at 24h for the same treatment. No isolated effect of the cultivar was observed for CAT activity, only the effects of treatment and time. In this case, the effect of the cultivar on CAT activity was observed only in the interaction C x HAI, being the only significant interaction among the others. However, CAT activity significantly decreased at 18h in the inoculated-R-PH 16 and tended to increase at 24h compared to its control. SOD activity was significantly influenced by the isolated effects of cultivar, treatment, and time, and by their interaction. Higher activity of this enzyme was observed in the control treatment at 6h and 24h and in the inoculated R-PH 16 at 8h and 24h. For the S-SJ 02, there were differences among the means, with the most significant contrasts observed in the control treatment at 12h and the inoculated treatment at 24h, which showed the lowest SOD activities.

**Figure 2:**
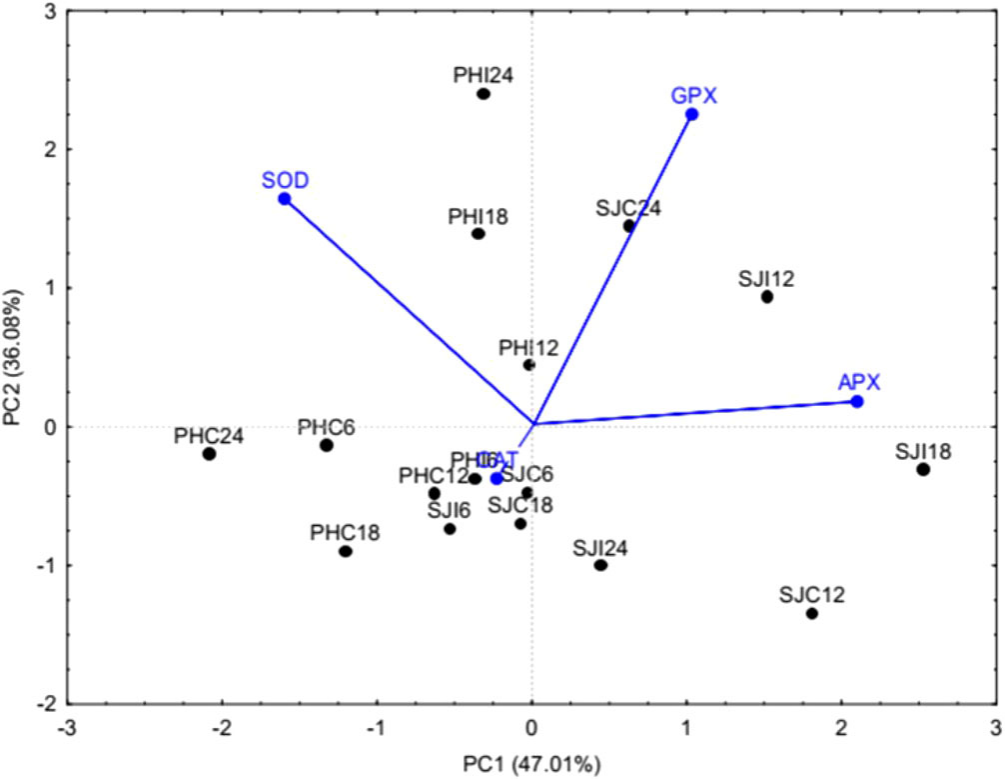
Biplot for oxidative stress enzyme activity in control and *P. citrophthora*-inoculated *Theobroma cacao* plants. APX, ascorbate peroxidase; GPX, guaiacol peroxidase; CAT, catalase; SOD, superoxide dismutase; SJ, susceptible cultivar (SJ 02); PH, resistant cultivar (PH 16); C, control; I, inoculated; 6, 6 hours after inoculation (HAI); 12, 12 hours after inoculation (HAI); 18, 18 hours after inoculation (HAI); 24, 24 HAI.

The principal component analysis summarized 83.09% of the total variance present in the data matrix. Specifically, PC1 explained 47.01% of the total variance, while PC2 accounted for 36.08% (**Figure 2**). The variable with the highest loading score in PC1 was APX, whereas in PC2, the highest loading score was observed for GPX. Therefore, the cumulative percentage of total variance in PC1 and PC2 was largely attributed to the variation stemming from APX and GPX, respectively. According to the correlation matrix derived from the PCA, GPX showed no correlation with SOD, and SOD did not correlate with CAT. The other correlations between variables were weak, either positive or negative.

The dispersion analysis showed that the treatments SJC12, SJC24, SJI12, SJI18, and SJI24 in the S-SJ 02 had a higher contribution to the percentage of total variance due to their greater distance from the origin of the graph. In the R-PH 16, a similar pattern was observed with treatments PHC6, PHC18, PHC24, PHI18, and PHI24, though at different times. In summary, all these treatments are considered contrasting compared to the others because of their higher association with APX activity, as seen in SJI18; higher association with GPX activity in SJC24, SJI12, PHI18, and PHI24; higher association with SOD activity in PHC6, PHC24, PHI18, and PHI24; lower association with SOD activity in SJC12 and SJI24; and higher association with CAT activity in PHC18. The other treatments showed a stable response, meaning that, due to their proximity to the origin of the graph, they contributed little to the total variance observed in the data matrix. High similarity in oxidative stress enzyme responses was observed among SJC6, SJC18, SJI6, PHC12, and PHI6.

### 3.2 Proteomic analysis

**Figure 3:**
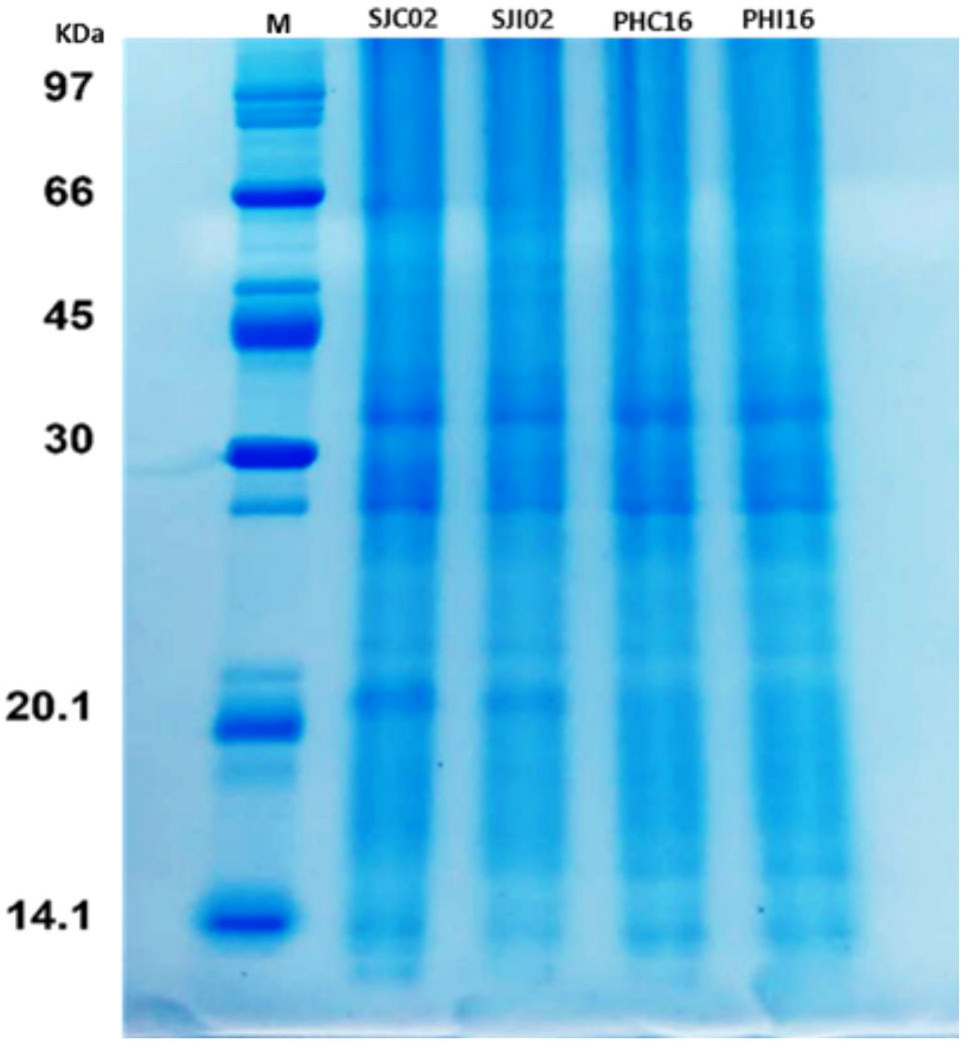
SDS-PAGE of total protein extract from *Theobroma cacao*, 24 hours post-inoculation with *P. citrophthora*. M, molecular weight marker; SJC02, control cultivar SJ 02; SHI02, inoculated cultivar SJ 02; PHC16, control cultivar PH 16; PHI16, inoculated cultivar PH 16. 40 μg of protein from each sample was loaded onto the gel.

The total protein extracts from the leaves of the clonal cultivars SJ 02 and PH 16, taken 24 hours after inoculation with *P. citrophthora*, as well as from the non-inoculated controls, were qualitatively assessed using an SDS-PAGE gel (**Figure 3**). A clear separation of bands (proteins) was observed across the range of 10 to 110 kDa, confirming that the material was of sufficient quality to proceed with LC-MS/MS analyses.

**Figure 4:**
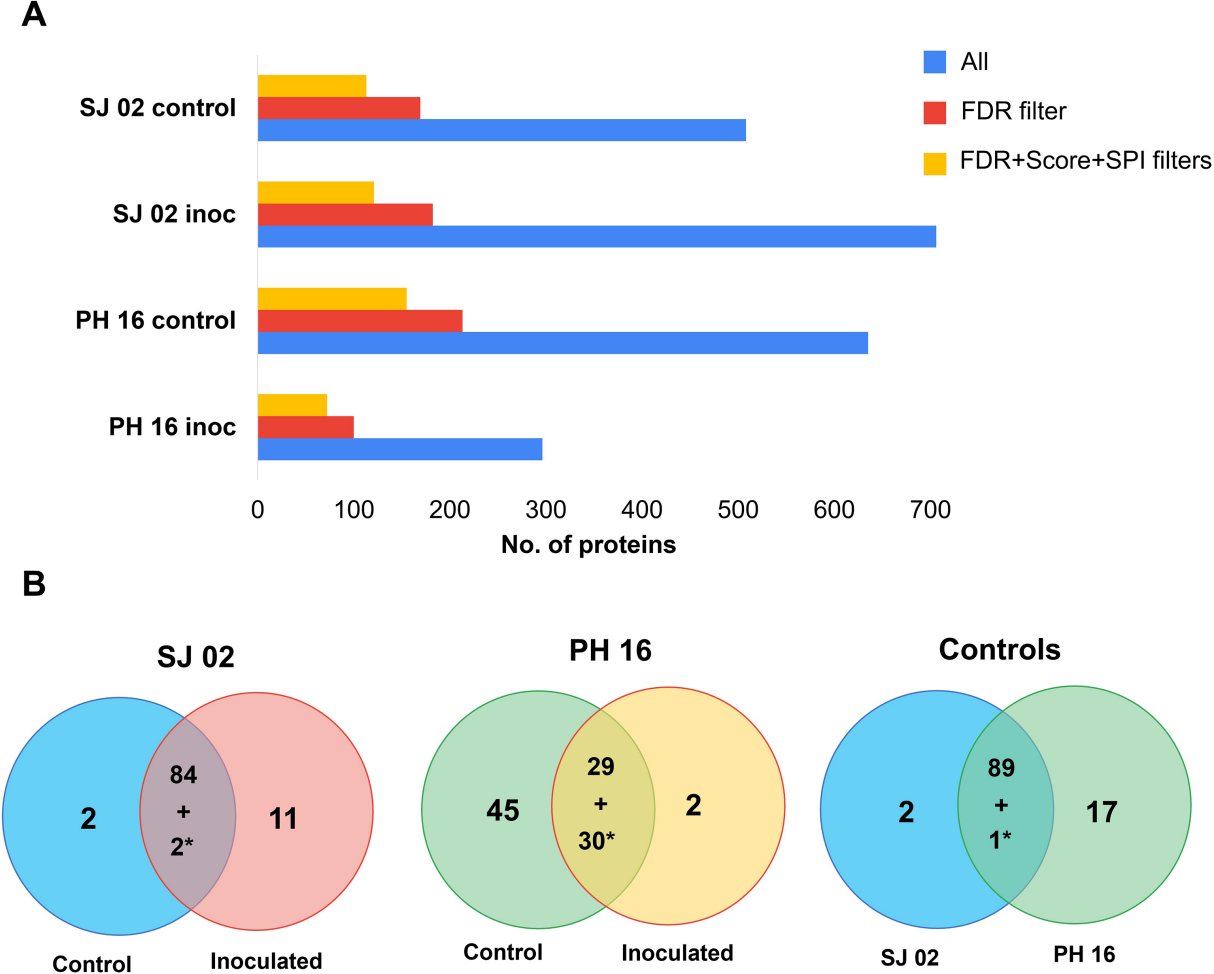
Proteins from *Theobroma cacao* leaves identified by LC-MS/MS. **(A)** Proteins identified in leaves from cultivars SJ 02 (susceptible) and PH 16 (resistant), both inoculated and control samples, following a search in the *T. cacao* protein database. All = all reported proteins after the database search. The proteins were first filtered by a false discovery rate (FDR)< 1%, followed by filters of score > 5 and Scored Peak Intensity (SPI) > 60%. **(B)** Proteins in at least two of the three technical replicates (Frequency > 60%) were analyzed using the Find Unique Entities method. The Venn diagrams show the number of unique and common proteins between treatments inoculated with *P. citrophthora* and the control (sprayed with sterile distilled water) in both susceptible and resistant cultivars, as well as between the controls of both cultivars. * = number of common proteins between treatments that meet the parameters of *p*-value ≤ 0.05 and fold change ≥ 1.5.

Through MS/MS analysis, 1,583 proteins were identified across the 4 treatments (susceptible and resistant cultivars; inoculated and control groups). Out of these, 205 proteins met the quality criteria of FDR<1%, Score > 5, and SPI > 60% (**Figure 4A**) and were considered “valid.”

Comparisons were made between the inoculated treatment and the control and between the controls of each cultivar (**Figure 4B**). Only proteins present in at least two technical replicates in one of the conditions were considered for comparison. Accordingly, 99 proteins from the treatments of SSJ 02, 72 proteins from the treatments of R-PH 16, and 109 proteins from the control treatments of both cultivars were subjected to unique entity analysis and T-test.

In the S-SJ 02, 85% of the compared proteins were common between the inoculated treatment and the control. Only 2 of these common proteins showed significant differential abundance (p-value ≤ 0.05; fold change ≤ 1.5). The inoculated treatment of the S-SJ 02 had five times more unique proteins than the inoculated treatment of the resistant cultivar, with the control condition showing the highest number of unique proteins among the three comparisons (**Figure 4B**). When comparing the control treatments, more than 80% of the proteins were present in both clones, with only one protein being differentially abundant. However, the control of the R-PH 16 had eight times more unique proteins than the control of the S-SJ 02 (**Figure 4B**).

We present the accumulation of unique and differentially abundant proteins in comparing the inoculated treatment and the control for each cultivar (**Tables 2**, **3**). Similarly, we show the unique and commonly differentially abundant proteins identified in the controls of *Theobroma cacao* cultivars PH 16 and SJ 02, according to the ShinyGO and UniProt tools (**Table 4**).

**Table 2:** Unique and common differentially abundant proteins identified in the susceptible *Theobroma cacao* cultivar SJ 02.

Protein ID^a^	Relative abund.^b^	Protein name	Mw (Da)^c^	pI^d^	Score^e^	Functional annotation^f^
A0A061DLY9	∞	Ribosomal protein L5 B isoform 1	40545.6	9.59	8.48	Traslation
A0A061GF50	∞	Malate dehydrogenase	37292.4	8.72	14.45	Carbohydrate metabolic process
A0A061GS01	∞	Mitochondrial substrate carrier family protein	32297.1	9.71	7.37	Malate transmembrane transport
A0A061EVX4	∞	Glutamate synthase 1 isoform 1	178315.3	5.99	8.87	Alpha-amino acid biosynthetic process
A0A061FDM1	∞	Isoflavone reductase-like protein 4 isoform 1	34885.5	6.15	8.73	Oxidoreductase activity
A0A061GBW3	∞	40S ribosomal protein SA	34405.4	5.04	10.83	Traslation
A0A061EMF9	∞	Malate dehydrogenase	36119	6.61	16.67	Carbohydrate metabolic process
A0A061F8V4	∞	Ribosomal protein S5/Elongation factor G/III/V family protein	95254.9	5.86	15.94	Traslation
A0A061GDW9	∞	NAD(P)-binding Rossmann-fold superfamily protein isoform 2	32256.3	7.79	10.46	Ubiquinone-6 biosynthetic process
E3VU17	∞	Cytochrome b6	24264.2	9.36	14.18	Photosynthesis
A0A061ETY3	∞	Glutaredoxin-dependent peroxiredoxin	24103.1	9.22	13.65	Response to oxidative stress
A0A061G750	0	NAD(P)-binding Rossmann-fold superfamily protein isoform 1	32169.2	7.79	23.07	Ubiquinone-6 biosynthetic process
A0A061GUD0	0	Lactoylglutathione lyase	42641.2	8.9	7.67	Lactate metabolic process
A0A061DYK6	↑	Fructose-bisphosphate aldolase	43305.7	8.75	113.41	Carbohydrate metabolic process
E3VU13	↑	Photosystem II CP47 reaction center protein	56283.3	6.27	182.6	Photosynthesis

a Protein ID corresponding to the *Theobroma cacao* proteome in the UniProt database (https://www.uniprot.org/).

b Relative protein abundance in the SJ 02 cultivar inoculated with *P. citrophthora* compared to its control (sprayed with sterile distilled water). ∞= unique to the inoculated treatment; 0= unique to the control treatment; ↑ = common differentially abundant (*p*-value< 0.05 and fold change≥ 1.5) with increased abundance in the inoculated treatment relative to the control.

c Molecular weight in daltons (Da).

d Isoelectric point.

e Reflects the information content (amount of useful fragmentation) in the MS/MS spectrum. Calculated by summing the scores of all peptides detected for the protein.

f Protein annotation according to analysis in the ShinyGO software and UniProt.

**Table 3:** Unique and common differentially abundant proteins identified in the resistant cultivar PH 16 of *Theobroma cacao*.

Protein ID^a^	Relative abund.^b^	Protein name	Mw (Da)^c^	pI^d^	Score^e^	Functional annotation^f^
A0A061ERE1	∞	Histone H2A	15785.3	10.59	66.66	Structural constituent of chromatin
A0A061FRA0	∞	Arginase	58990.8	5.97	9.08	Alpha-amino acid metabolic process
E3VTW8	↓	Photosystem II protein D1	39033.9	5.12	52.13	Photosynthesis
A0A061EKX7	↓	Photosystem I subunit D-2	23298.3	9.67	26.3	Photosynthesis
A0A061FHZ0	↓	Chlorophyll a-b binding protein, chloroplastic	31158.1	5.74	173.3	Photosynthesis
A0A061ERA1	↓	Histone H4 (Fragment)	16128.5	11.33	24.88	Structural constituent of chromatin
A0A061G7Z6	↓	Phosphoribulokinase	45855.3	6.23	15.58	Carbohydrate metabolic process
E3VTZ5	↓	Ribulose bisphosphate carboxylase large chain	54378.8	6.04	345.07	Carbohydrate metabolic process
E3VTY6	↓	Photosystem I P700 chlorophyll a apoprotein A2	82516.6	6.71	78.07	Photosynthesis
E3VU13	↓	Photosystem II CP47 reaction center protein	56283.3	6.27	108.17	Photosynthesis
A0A061DG44	↓	Photosystem II subunit O-2	35385.3	5.85	80.9	Photosynthesis
A0A061EQW6	↓	Chlorophyll a-b binding protein, chloroplastic	31040.9	5.66	38.54	Photosynthesis
E3VTY3	↓	Photosystem II D2 protein	39776.9	5.33	48.96	Photosynthesis
A0A061DSX4	↓	Photosystem II subunit P-1	28683.7	8.92	140.07	Photosynthesis
A0A061EH79	↓	Ribulose bisphosphate carboxylase small chain	21187.1	9.63	120.73	Carbohydrate metabolic process
E3VTY4	↓	Photosystem II CP43 reaction center protein	52026	6.68	70.71	Photosynthesis
E3VTZ4	↓	ATP synthase subunit beta	53725.6	5.29	182.46	ATP metabolic process
A0A061GD38	↓	Chlorophyll a-b binding protein, chloroplastic	28236.6	5.13	121.36	Photosynthesis
A0A061ECJ8	↓	Glyceraldehyde-3-phosphate dehydrogenase	36878.8	8.7	91.1	Carbohydrate metabolic process
E3VTX3	↓	ATP synthase subunit alpha	55369.1	5.19	132.14	ATP metabolic process
A0A061EDL4	↓	Glyceraldehyde-3-phosphate dehydrogenase	43281.5	8.49	72.55	Carbohydrate metabolic process
A0A061FB18	↓	Phosphoglycerate kinase	51478.4	8.67	146.41	Carbohydrate metabolic process
A0A061GWC1	↓	Superoxide dismutase [Cu-Zn]	22950.8	6.34	82.45	Response to oxidative stress
A0A061GK99	↓	Germin-like protein	29774.4	9.86	27.18	Manganese ion binding
A0A061FHB6	↓	Chlorophyll A-B binding family protein	29097.4	8.8	39.88	Photosynthesis
A0A061F5T3	↓	rRNA N-glycosidase	309600.7	5.19	9.73	Response to stress
E3VTY7	↓	Photosystem I P700 chlorophyll a apoprotein A1	83326.2	6.67	107.27	Photosynthesis
A0A061GD16	↓	PSI-F	24809.4	9.75	83.79	Photosynthesis
A0A061FDN2	↓	Plastocyanin	16840.8	4.79	58.64	Generation of precursor metabolites and energy
A0A061FA06	↓	(S)-2-hydroxy-acid oxidase	40826.6	9.41	62.52	Photorespiration
A0A061GEN3	↓	Larreatricin hydroxylase	67890.1	5.96	20.01	Pigment biosynthetic process
A0A061DYK6	↓	Fructose-bisphosphate aldolase	43305.7	8.75	123.57	Carbohydrate metabolic process
A0A061F6X3	0	Chloroplastic lipocalin	38830.7	5.78	5.97	Response to toxic substance
H6S166	0	Dehydroascorbate reductase	23710.1	5.99	6.66	Cellular oxidant detoxification
A0A061F2D5	0	GDP-mannose 3,5-epimerase 1	48502.6	6.89	12.35	GDP-mannose 3,5-epimerase activity
A0A061EFZ7	0	Dehydroascorbate reductase 1 isoform 1	29395.3	6.6	10.34	Response to oxidative stress
A0A061E2I2	0	Nucleoside diphosphate kinase	16506.7	6.31	18.94	GTP biosynthetic process
A0A061ESD6	0	Aquaporin TIP1,6	26007.6	6.17	15.19	Water transport
A0A061DFA2	0	Ferredoxin--NADP reductase, chloroplastic	41711.3	9.09	25.95	Ferredoxin-NADP+ reductase activity
A0A061DLD6	0	Polyubiquitin 10	51190.8	7.05	20.59	Protein modification
A0A061E7Y6	0	Protein SDA1	91882.8	6.06	7.28	Ribosome localization
A0A061GHX3	0	Aminomethyltransferase	44735.5	9.18	16.74	Alpha-amino acid metabolic process
A0A061F1U6	0	Superoxide dismutase [Cu-Zn]	25185.9	5.67	13.97	Response to oxidative stress
A0A061GTT9	0	Alanine--glyoxylate aminotransferase	44647.7	7.84	29.24	Alpha-amino acid metabolic process
A0A061GKZ9	0	Thioredoxin-dependent peroxiredoxin	23996.9	9.59	17.15	Response to oxidative stress
A0A061DX77	0	Malate dehydrogenase	41750.7	9.43	17.67	Carbohydrate metabolic process
A0A061EDH0	0	ATP binding,ATP-dependent helicases,DNA helicases	142569.3	6.58	7.33	RNA catabolic process
A0A061E463	0	Photosystem II stability/assembly factor, chloroplast (HCF136) isoform 1	45284.2	7.88	17.7	Photosynthesis
A0A061GBW3	0	40S ribosomal protein SA	34405.4	5.04	5.47	Translation
A0A061F8V4	0	Ribosomal protein S5/Elongation factor G/III/V family protein	95254.9	5.86	9.65	Translation
A0A061F1M8	0	Photosystem I subunit E-2-like protein	19667	9.62	8.26	Photosynthesis
A0A061GJ52	0	Cell division protease ftsH isoform 1	107944.7	6.74	8.18	Proteolysis
A0A061E4X1	0	Inorganic diphosphatase	36719.2	6.94	12.68	Phosphate-containing compound metabolic process
A0A061E7U5	0	Cyclase family protein	31124.4	5.6	15.89	Alpha-amino acid metabolic process
A0A061FUY8	0	Elongation factor Tu	52403.2	6.45	35.41	Translation
A0A061G750	0	NAD(P)-binding Rossmann-fold superfamily protein isoform 1	32169.2	7.79	17.94	Ubiquinone-6 biosynthetic process
A0A061FNK9	0	2-oxoglutarate dehydrogenase, E1 component	118249.6	6.53	8.52	Cellular respiration
A0A061EAM9	0	SPFH/Band 7/PHB domain-containing membrane-associated protein family isoform 1	31730.6	5.31	22.91	Protein histidine kinase binding
A0A061G4F3	0	Pentose-5-phosphate 3-epimerase	30147.4	9.02	27.67	Carbohydrate metabolic process
A0A061FW44	0	Uncharacterized protein	20364.3	5.54	11.56	Transmembrane protein
A0A061F263	0	Thylakoid lumen 18.3 kDa protein	32333.3	8.51	15.15	Membrane protein
A0A061F909	0	Photosystem II 10 kDa polypeptide, chloroplastic	14210	9.73	30.32	Photosynthesis
A0A061F3W4	0	Chloroplast RNA binding	42743.1	8.84	14.45	Ribosome biogenesis
A0A061DIX8	0	Serine hydroxymethyltransferase	57673.2	8.98	38.79	Alpha-amino acid metabolic process
A0A061G4C2	0	Chlorophyll a-b binding protein, chloroplastic	28048.6	8.98	33.5	Photosynthesis
A0A061H075	0	Uncharacterized protein isoform 1	92415.3	5.48	14.39	Unannotated
A0A061FJ08	0	Arginase isoform 1	37134.7	6.09	41.31	Alpha-amino acid metabolic process
E3VU00	0	Cytochrome f	35278	7.79	34.22	Photosynthesis
A0A061FWL5	0	21 kDa seed protein	24278.9	5.71	20.17	Negative regulation of peptidase activity
A0A061GRY8	0	Glycine cleavage system P protein	114652.1	6.89	13.12	Alpha-amino acid metabolic process
A0A061FNG9	0	Chlorophyll a-b binding protein, chloroplastic	26817.3	6.53	14.64	Photosynthesis
A0A061E2I3	0	Chlorophyll a-b binding protein, chloroplastic	27176.7	8.8	49.74	Photosynthesis
A0A061E3S5	0	Plastoquinol--plastocyanin reductase	24688.9	8.8	27.41	Photosynthesis
A0A061GG32	0	Plastid-lipid associated protein PAP/fibrillin family protein	47554.7	8.47	30.73	Membrane protein
A0A061ECF1	0	Uncharacterized protein	92561	6.43	11.45	Phloem development
A0A061FLN9	0	Chlorophyll a-b binding protein, chloroplastic	30316.2	6.34	28.63	Photosynthesis
A0A061GY51	0	Histone H2A	15785.3	10.59	66.66	Structural constituent of chromatin

a Protein ID corresponding to the *Theobroma cacao* proteome in the UniProt database (https://www.uniprot.org/).

b Relative abundance of the protein in the PH 16 cultivar inoculated with *P. citrophthora* compared to its control (sprayed with sterile distilled water). ∞ = exclusive to the inoculated treatment; 0 = exclusive to the control treatment; ↓ = commonly differentially abundant (p-value< 0.05 and fold change ≥ 1.5) with decreased abundance in the inoculated treatment relative to the control.

c Molecular weight in Daltons (Da).

d Isoelectric point.

e Reflects the content of information (amount of useful fragmentation) in the MS/MS spectrum. Calculated by summing the scores of all peptides detected for the protein.

f Protein annotation according to analysis in ShinyGO and UniProt software.

**Table 4:** Proteins exclusively and differentially abundant in the controls of PH 16 and SJ 02 cultivars of *Theobroma cacao*.

Protein ID^a^	Relative abund.^b^	Protein name	Mw (Da)^c^	pI^d^	Score^e^	Functional annotation^f^
A0A061EVI6	↑	Thioredoxin-dependent peroxiredoxin*	29447.1	7.83	53.64	Response to oxidative stress
A0A061DV22	0	Cysteine synthase	41892.1	8.97	11.12	Alpha-amino acid metabolic process
A0A061GNN2	0	3-hydroxyacyl-CoA dehydrogenase	78959.9	9.48	9.16	Lipid metabolism
A0A061EAM9	∞	SPFH/Band 7/PHB domain-containing membrane-associated protein family isoform 1	31730.6	5.31	22.91	Protein histidine kinase binding
E3VU17	∞	Cytochrome b6*	24264.2	9.36	19.89	Photosynthesis
A0A061E7U5	∞	Cyclase family protein	31124.4	5.6	15.89	Alpha-amino acid metabolic process
A0A061H075	∞	Uncharacterized protein isoform 1	92415.3	5.48	14.39	Unannotated
A0A061FW44	∞	Uncharacterized protein	20364.3	5.54	11.56	Transmembrane protein
A0A061ECF1	∞	Uncharacterized protein	92561	6.43	11.45	Phloem development
A0A061F8V4	∞	Ribosomal protein S5/Elongation factor G/III/V family protein	95254.9	5.86	9.65	Translation
A0A061F1M8	∞	Photosystem I subunit E-2-like protein	19667	9.62	8.26	Photosynthesis
A0A061GJ52	∞	Cell division protease ftsH isoform 1	107944.7	6.74	8.18	Proteolysis
H6S166	∞	Dehydroascorbate reductase	23710.1	5.99	6.66	Response to oxidative stress
A0A061F6X3	∞	Chloroplastic lipocalin	38830.7	5.78	5.97	Response to toxic substance
A0A061GBW3	∞	40S ribosomal protein SA	34405.4	5.04	5.47	Translation
A0A061E463	∞	Photosystem II stability/assembly factor, chloroplast (HCF136) isoform 1	45284.2	7.88	17.7	Photosynthesis
A0A061ESD6	∞	Aquaporin TIP1,6	26007.6	6.17	15.19	Water transport
A0A061F2D5	∞	GDP-mannose 3,5-epimerase 1	48502.6	6.89	12.35	GDP-mannose 3,5-epimerase activity
A0A061EFZ7	∞	Dehydroascorbate reductase 1 isoform 1	29395.3	6.6	10.34	Response to oxidative stress
A0A061E7Y6	∞	Protein SDA1	91882.8	6.06	7.28	Ribosome localization

a Protein ID corresponding to the *Theobroma cacao* proteome in the UniProt database (https://www.uniprot.org/).

b Relative abundance of the protein in the PH 16 cultivar control compared to the SJ 02 cultivar control. **∞**= exclusive to the PH 16 cultivar control; 0 = exclusive to the SJ 02 cultivar control; ↑ = commonly differentially abundant (p-value< 0.05 and fold change≥ 1.5) with increased abundance in the PH 16 cultivar compared to the SJ 02 cultivar.

c Molecular weight in daltons (Da).

d Isoelectric point.

e Reflects the information content (amount of useful fragmentation) in the MS/MS spectrum. Calculated by summing the scores of all detected peptides for the protein.

f Protein annotation according to analysis in ShinyGO and UniProt.

*Proteins that did not show significant differences in abundance between the control and inoculated treatment of the PH 16 cultivar.

**Figure 5:**
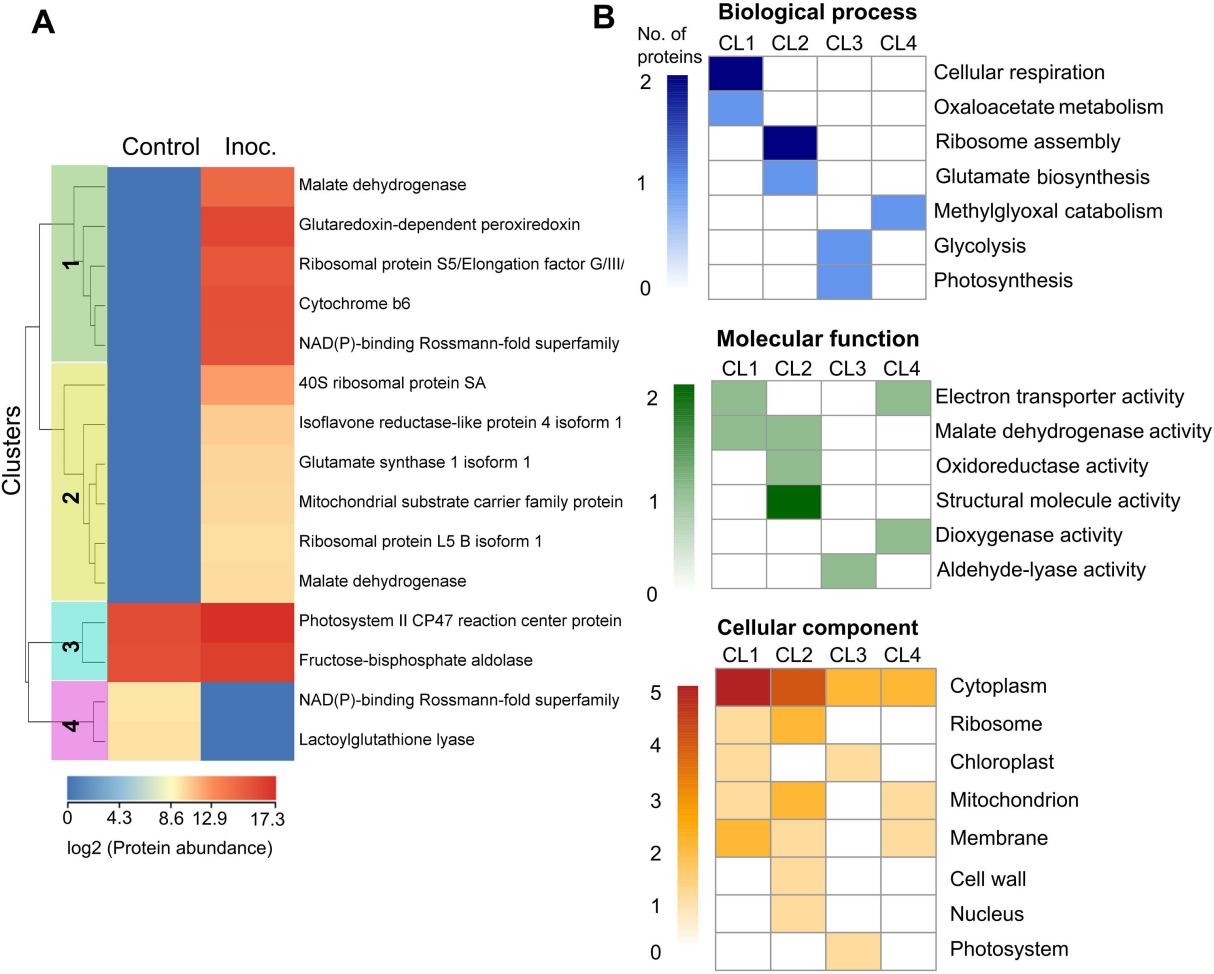
Clustering Heatmap and Functional Enrichment Analysis. The clustering heatmap **(A)** illustrating the abundance and **(B)** functional enrichment of unique and common differentially abundant proteins identified in the comparison between the *P. citrophthora*-inoculated treatment and the control of the susceptible cultivar SJ 02.

The clustering analysis of exclusive and differentially abundant proteins identified in the S-SJ 02revealed 4 clusters corresponding to the protein abundance patterns (**Figure 5A**). Cluster 1 contains proteins unique to the inoculated treatment found in higher abundance. In this cluster, cellular respiration was the most enriched biological process (**Figure 5B**), with electron transport and malate dehydrogenase activity being the most represented molecular functions. Most of these proteins are located in the cytoplasm and cell membranes. Cluster 2 shows proteins of lower abundance that are unique to the inoculated treatment. The most represented process in this treatment was ribosome assembly. Additionally, oxidoreductase activity and malate dehydrogenase activity were enriched molecular functions in this cluster, with most of the proteins located in the cytoplasm, ribosome, and mitochondria (**Figure 5B**). Cluster 3 corresponds to two proteins common to both treatments, which showed increased abundance in the inoculated treatment. These proteins are involved in photosynthesis and glycolysis processes (**Figure 5B**). Finally, cluster 4 includes proteins unique to the control treatment, one of which is involved in the catabolism of methylglyoxal.

**Figure 6:**
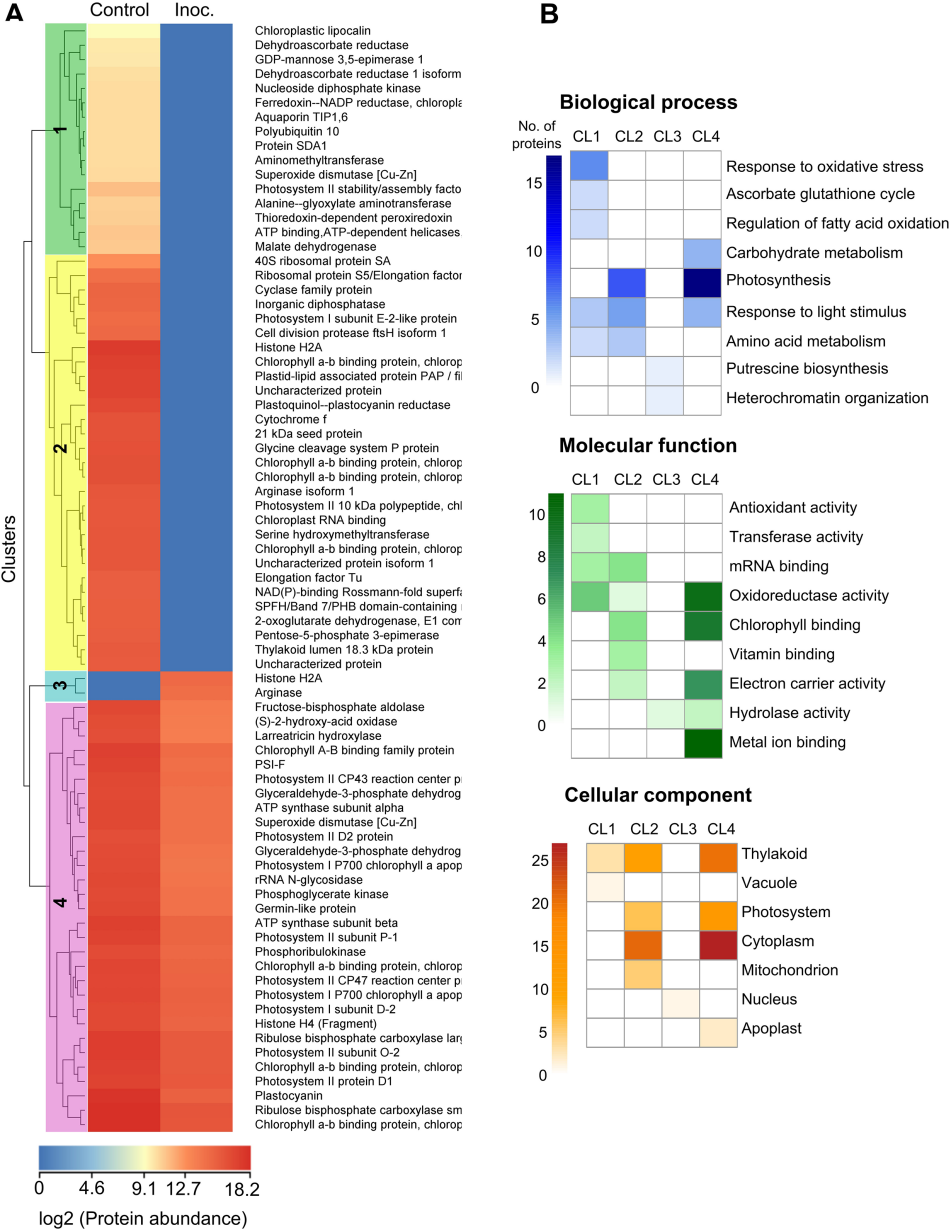
Clustering heatmap and functional enrichment analysis. Clustering heatmap **(A)** illustrating the abundance and **(B)** functional enrichment of unique and common differentially abundant proteins identified in the comparison between the *P. citrophthora*-inoculated treatment and the control of the resistant cultivar PH 16.

Four clusters were established in the clustering analysis of the resistant cultivar’s differentially abundant exclusive and common proteins based on the protein abundance profile (**Figure 6A**). Cluster 1 contains proteins unique to the control treatment, which are present in lower abundance (**Figure 6A**). The most enriched process in this group is the response to oxidative stress, with the most represented molecular function and cellular component being oxidoreductase activity and the thylakoid, respectively. In Cluster 2, which includes proteins exclusive to the control treatment with higher abundance, photosynthesis is the most represented biological process. The most represented molecular functions are mRNA and chlorophyll-binding, while the most represented cellular components are the cytoplasm and thylakoid. The group of proteins exclusive to the inoculated treatment (Cluster 3) represents processes such as putrescine biosynthesis and heterochromatin organization. In Cluster 4, proteins common to both treatments but showing decreased abundance in the inoculated treatment are associated with photosynthesis and carbohydrate metabolism processes, along with oxidoreductase activity and binding to chlorophyll and metals.

**Figure 7:**
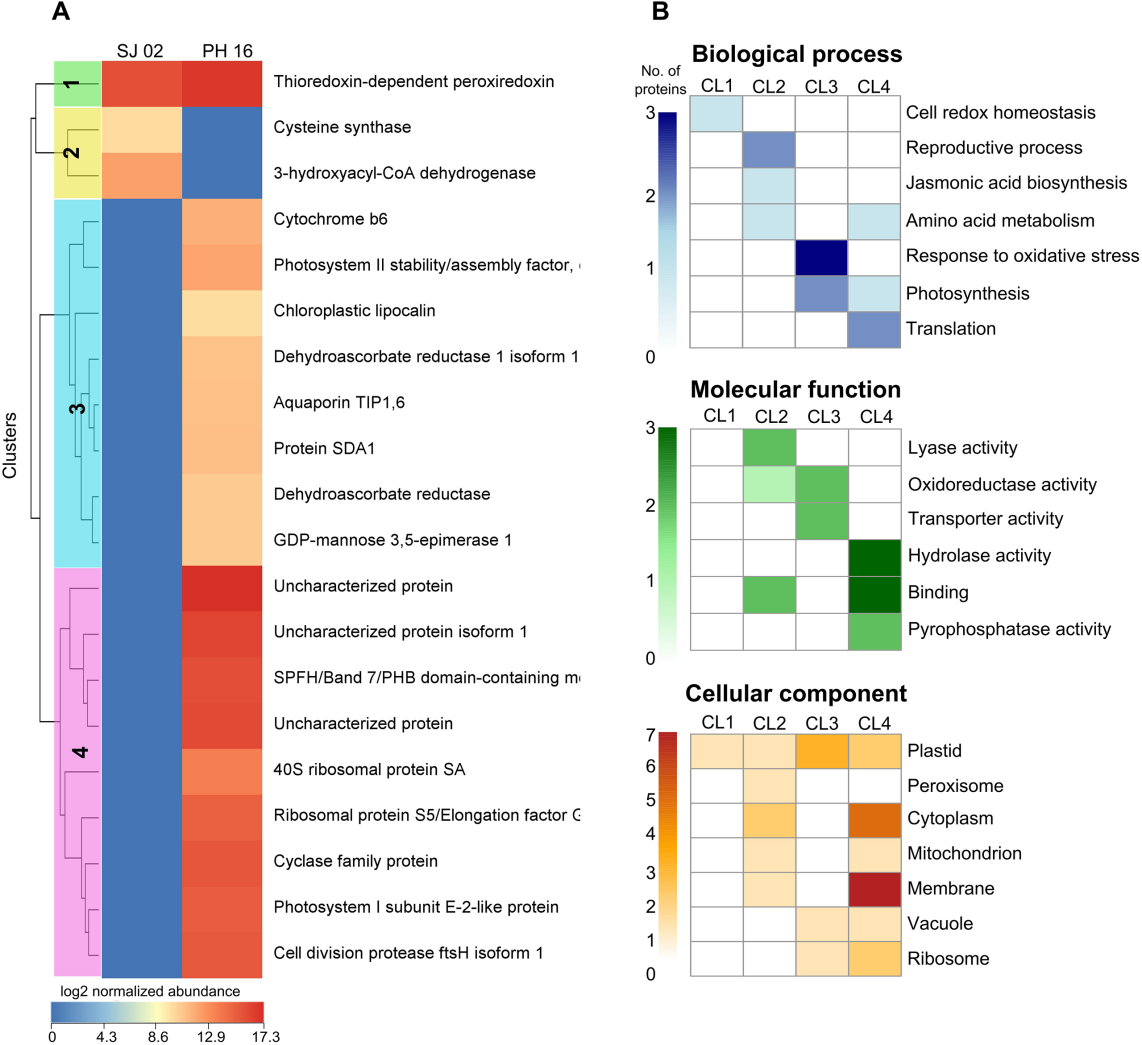
Clustering heatmap and functional enrichment analysis. **(A)** Clustering heatmap illustrating the abundance and **(B)** functional enrichment of unique and common differentially abundant proteins identified in the comparison between the controls (inoculated with sterile distilled water) of the resistant cultivar (PH 16) and the susceptible cultivar (SJ 02).

The clustering analysis comparing the controls of the cultivars revealed four clusters (**Figure 7A**). Cluster 1 consists of a protein with higher abundance in the R-PH 16, which is involved in the cell’s redox homeostasis process (**Figure 7B**). Cluster 2 includes two proteins unique to the control of the S-SJ 02, which are involved in the reproductive process. The proteins unique to the control treatment of the R-PH 16 are found in clusters 3 and 4. The most represented process in Cluster 3 is the response to oxidative stress, while in Cluster 4, it is protein translation.

**Figure 8:**
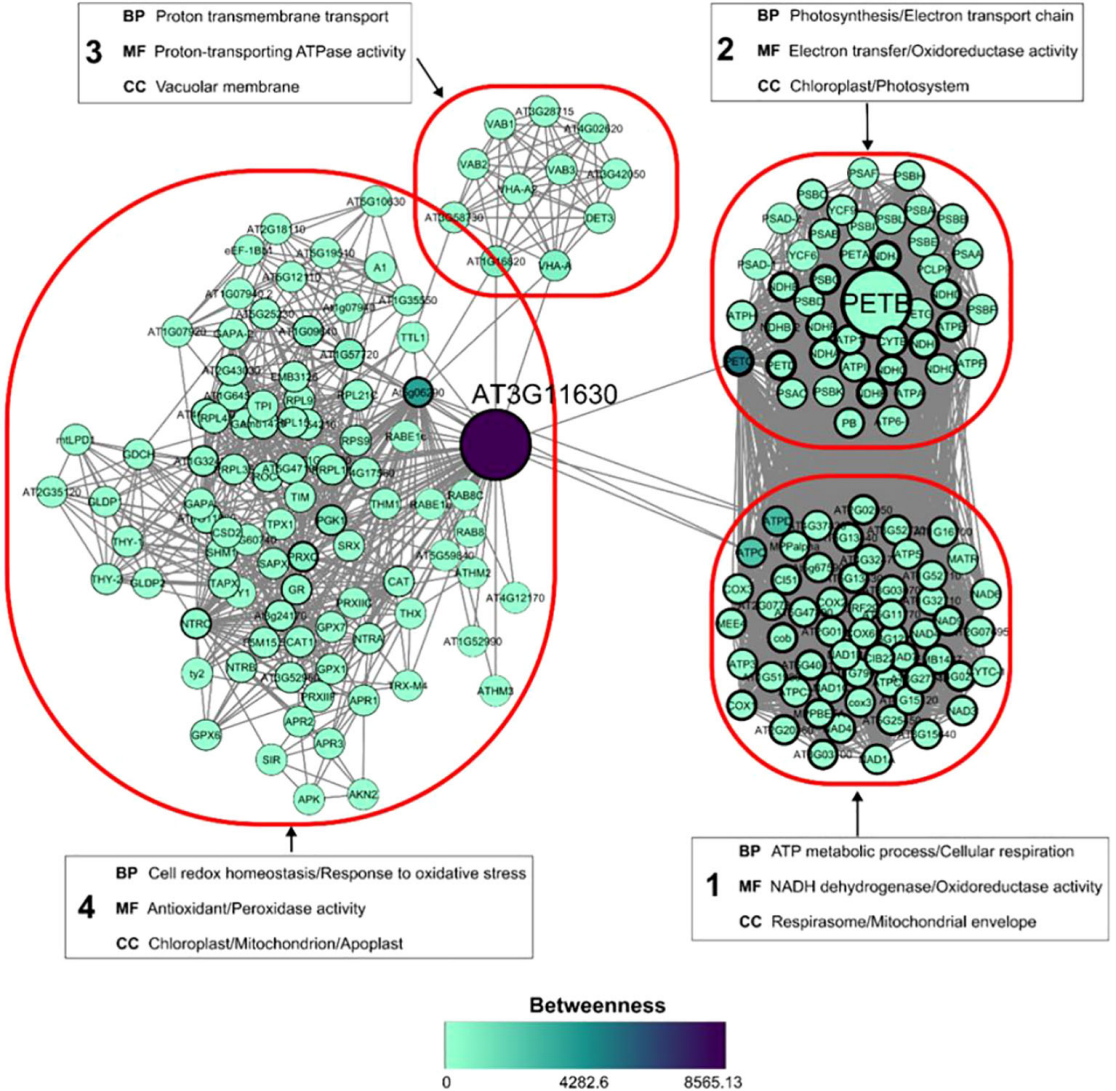
Protein-protein interaction network. Interaction of *Arabidopsis thaliana* proteins based on orthologous proteins identified in *T. cacao* (larger nodes), which are more abundant in the control treatment of the resistant cultivar to *P. citrophthora*, compared to the control treatment of the susceptible cultivar, and showed no difference in abundance in pathogen-inoculated seedlings. The edge width of the nodes represents the node degree parameter, and the node color represents the Betweenness value. Red circles outline the network clusters, and in each cluster, the functional enrichment corresponding to biological process (BP), molecular function (MF), and cellular component (CC) was analyzed.

Among the proteins more abundant in the resistant genotype control, a thioredoxin-dependent peroxiredoxin and Cytochrome b6 were more abundant in the resistant control than in the susceptible one. However, the abundance of these two proteins did not change in the treatments inoculated with *P. citrophthora* (**Table 4**; **Figure 7**). An interaction network was constructed from orthologs of these two proteins in *Arabidopsis thaliana* to understand their role in plant defense (**Figure 8**). The network comprises 202 nodes (proteins), 3660 connectors, and 4 clusters. The protein with the highest betweenness centrality was the ortholog of the thioredoxin-dependent peroxiredoxin, while the protein with the highest degree centrality was the ortholog of Cytochrome b6.

## 4 Discussion

The two cocoa cultivars used in this work exhibited distinct enzymatic activity and proteomic profiles upon inoculation with *P. citrophthora*. To a large extent, these differences can be attributed to differences in response patterns to infection by the pathogen, as they contrast in their resistance to it. PH 16 and SJ 02 are modern Brazilian cocoa cultivars (Santos et al., 2015), contrasting in genetic diversity (Bertolde et al., 2010), and in their resistance to black pod rot (Lopez et al., 2006). Both cultivars are products of complex crosses involving germplasm from multiple original genetic groups, a typical approach in modern breeding programs aimed at combining desirable traits. Specifically, S-SJ 02 exhibits ancestry primarily from the Contamana (approx. 40.7%), Iquitos (34.5%), and Amelonado (23.5%) genetic groups, as well as Criollo, Marañon, Nacional, and Purus, in smaller proportions (Freitas et al., 2025). Different sets of genes contribute to plant defense against pathogen, and population-specific transcriptional patterns of these genes were shaped by historical selective pressures, demonstrating how cacao genotypes have adapted to their local microbial environments in ways that influence their defensive capacities (Winters et al., 2024). In this context, the differentiated accumulation patterns of proteins and enzymes reported in the present work likely reflect both host genetic/evolutionary uniqueness and pathogen-induced differences during the interaction, as discussed below.

### 4.1 Management of ROS could be a factor in the resistance of cacao trees to Black pod

Differences in the activity of enzymes involved in oxidative stress and the abundance of proteins were observed in the leaves of *Theobroma cacao* cultivars, which vary in their resistance to the Black pod. These differences were noted when the cultivars were inoculated with *P. citrophthora* compared to their controls (seedlings inoculated with distilled water).

Superoxide dismutases (SODs) form the first line of defense against reactive oxygen species by catalyzing the dismutation of superoxide radicals (O^-2^) into oxygen and hydrogen peroxide (H_2_O_2_) (Alscher et al., 2002). The increase in SOD enzymatic activity was observed in the inoculated treatments at 12 HAI in the S-SJ 02 and at 18 HAI in the R-PH 16, compared to their respective controls (**Figure 1**). This suggests that oxidative stress occurred earlier in the S-SJ 02 than in the R-PH 16.

The conversion of H_2_O_2_ into water is catalyzed by the enzymes APX, GPX, and CAT (Kalisz et al., 2019). The peak activity of APX at 18 HAI in the S-SJ 02 was twice as high as that of the R-PH 16, indicating that oxidative stress is likely higher in the susceptible cultivar. Excessive production of H_2_O_2_ can cause oxidative damage to macromolecules and cellular structures. However, at non-toxic levels, this molecule plays a central role in stress signal transduction pathways (Hossain et al., 2015). Interestingly, in the R-PH 16, proteins related to the ascorbate-glutathione cycle, the main H_2_O_2_ detoxification system (Caverzan et al., 2012), decreased in abundance under the inoculated treatment (**Table 3**; H6S166, A0A061EFZ7). Better management of H_2_O_2_ production might have reduced cellular damage while allowing this molecule to act as a stress signal.

Furthermore, a glutaredoxin-dependent peroxiredoxin was one of the most abundant proteins in the inoculated treatment of the S-SJ 02. This enzyme eliminates ROS, specifically H_2_O_2_ (Rouhier et al., 2002), suggesting a high compound level at 24 HAI.

### 4.2 Contrasting expression of malate dehydrogenases in resistant and susceptible cultivars induces metabolic changes related to the response to *P. citrophthora*

In the S-SJ 02, only 15 proteins showed changes in abundance in the inoculated treatment compared to its non-inoculated control, with most being unique to or increasing in abundance in the inoculated treatment (**Figure 4B**). This suggests a low level of proteomic alteration 24 hours after inoculation in the S-SJ 02. The most represented processes by these proteins were the metabolism of small molecules such as malate, glutamate, and fructose (**Table 2**; **Figure 5B**).

A lactoylglutathione lyase, or glyoxalase (GLX), was identified only in the control treatment, indicating a decrease caused by *P. cirtrophthora* infection. This enzyme plays a crucial role in detoxifying methylglyoxal (MG) MG, a byproduct of metabolism that, under normal conditions, is detoxified and maintained at low concentrations within the cell. However, MG can reach toxic levels when the plant is under stress (Hoque et al., 2016). Therefore, it can be suggested that the plant is likely being affected by the accumulation of this cytotoxic compound.

In the treatment where the S-SJ 02 was inoculated, two malate dehydrogenases (MDH) were identified as exclusive to this treatment (**Table 2**). MDH catalyzes the reversible conversion of malate to oxaloacetate in the tricarboxylic acid cycle (Zhang and Fernie, 2018). Overexpression of an MDH in alfalfa and tobacco has resulted in higher organic acid production and increased aluminum tolerance (Tesfaye et al., 2001; Wang et al., 2010).

In tomatoes, the suppression of malate dehydrogenase is associated with increased starch accumulation, photosynthetic rate, and ascorbate levels (Nunes-Nesi et al., 2005). An inverse correlation between MDH and ascorbate was observed in Arabidopsis (Tomaz et al., 2010). In maize, MDH has shown its role in regulating the balance between mitochondrial respiration, glycolysis, and ATP production (Chen et al., 2020). In a cacao cultivar resistant to *P. citrophthora*, a malate dehydrogenase was identified only in the control, indicating a decrease in this protein as a response to the pathogen at 24 HAI (**Figure 6**). This suggests significant metabolic changes occurring.

### 4.3 The collapse of photosynthesis in the cacao tree might be a factor in resistance to *P. citrophthora*

Overall, the R-PH 16 exhibited a decrease in the abundance of all identified proteins in seedlings 24 hours after infection with *P. citrophthora* (**Table 3**; **Figure 6A**). Photosynthesis was the most represented process among the proteins that decreased in abundance, suggesting that this might have been one of the first processes affected by the pathogen. In cocoa leaves inoculated with *P. palmivora*, defense proteins with increased accumulation were identified 48 hours after inoculation. Additionally, several proteins involved in photosynthesis were found to be exclusive to the control treatment, indicating a significant decrease in the inoculated seedlings (Rego et al., 2022), which is consistent with the findings of this study with *P. citrophthora*.

In resistant tobacco leaves infected with *Phytophthora nicotianae*, photosynthesis collapse occurred 6 HAI and preceded hypersensitive cell death (Scharte et al., 2005). In this case, the authors concluded that the decline in photosynthesis was a prerequisite to triggering processes such as a metabolic shift towards non-assimilatory carbohydrate consumption, which initiated the defense response. With this information, it is possible to suggest that the same process may have occurred in the resistant *T. cacao* cultivar infected with *P. citrophthora* since the infected seedlings exhibited smaller hypersensitive response lesions than the S-SJ 02.

### 4.4 The jasmonic acid signaling pathway can be activated in the resistant cultivar after inoculation with *P. citrophthora*

Only one arginase was identified as unique in the treated inoculation of the R-PH 16 (**Table 3**). Arginase is involved in the synthesis of putrescine. This polyamine plays a role in responding to and tolerating various stresses by modulating cellular homeostasis, eliminating free radicals, regulating ABA levels, and preventing lipid peroxidation, among other functions (González-Hernández et al., 2022; Saha et al., 2015). It has been observed that *P. palmivora* significantly increases putrescine levels in oil palms during advanced stages of the disease compared to healthy plants (Moreno-Chacón et al., 2013).

In tomato leaves, both gene expression and the activity of an arginase were induced in response to wounding and treatment with jasmonic acid (JA), a potent signaling molecule for plant defense responses (Chen et al., 2004). In Arabidopsis plants treated with methyl jasmonate, there was an increase in the expression of genes involved in defense responses and oxidative stress. However, there was a decrease in the expression of genes related to chlorophyll constitution and photosynthesis (Jung et al., 2007). This information aligns with the protein profile observed in the resistant cocoa cultivar. It suggests that the JA pathway was activated in the early hours of infection with *P. citrophthora*, resulting in a decrease in proteins related to photosynthesis and an increase in the abundance of the arginase isoform. This, in turn, produces polyamines that help maintain the redox balance of cells (Saha et al., 2015).

### 4.5 preformed defenses as a resistance factor in R-PH 16

Preformed defenses are non-inducible, constitutive biochemical structures or mechanisms that exist before pathogen colonization in the plant (Belete, 2022). It was observed that cellular redox homeostasis and oxidative stress response were processes represented by the proteins present in the R-PH 16 not inoculated with the fungus.

Additionally, two proteins found in greater abundance in the R-PH 16 compared to the S-SJ 02, both non-inoculated, did not show a difference in abundance in the resistant inoculated cultivar (**Table 4**; **Figure 7A**). This identifies them as key proteins in non-induced resistance. One of these proteins was a thioredoxin-dependent peroxiredoxin involved in redox balance and oxidative stress response; the other was Cytochrome b6, involved in photosynthesis and cellular respiration.

The interaction network constructed from the orthologs of these two proteins (2-Cys peroxiredoxin BAS1 and a component of the cytochrome b6-f complex, respectively) reveals that cellular respiration, photosynthesis, and redox balance are undoubtedly connected through interactions established by these proteins (**Figure 8**). The orthologous protein to thioredoxin-dependent peroxiredoxin is directly linked to the cluster of proteins involved in photosynthesis through its connection with the PETC protein, which is a subunit of the cytochrome b6-f complex, an essential protein for photoautotrophy that provides resistance to photo-oxidative damage (Munekage et al., 2002). This network interaction is based on the co-expression of these proteins in *A. thaliana*, as the same transcription factor is responsible for the expression of these genes (Shaikhali et al., 2008).

One of the clusters identified is associated with cellular respiration occurring in the mitochondria. This cluster has more connections to the 2-Cys peroxiredoxin BAS1 than the cluster related to photosynthesis. This aligns with previous discussions about the metabolic shift towards non-assimilatory (heterotrophic) carbohydrate consumption, which triggers the defense response (Scharte et al., 2005). Therefore, when photosynthesis fails, the plant likely continues using the mitochondrial respiration pathway to obtain energy during the hypersensitive response while expressing Cytochrome b6 and thioredoxin-dependent peroxiredoxin to protect the plant from oxidative damage.

Two dehydroascorbate reductases (DHARs) were exclusively found in the control of the R-PH 16 (**Tables 3**, **4**; H6S166, A0A061EFZ7). DHARs are key enzymes in the ascorbic acid (AsA) recycling system, reducing dehydroascorbate (DHA) back to AsA. Additionally, they play a role in maintaining redox homeostasis by eliminating reactive oxygen species (ROS) under oxidative stress (Dixon et al., 2002).

However, DHARs were not identified in the R-PH 16 inoculated at 24 HAI. DHARs play a crucial role in maintaining AsA levels in chloroplasts. Therefore, the decrease in DHARs suggests an increase in DHA that is not being converted back to AsA. This shift in the AsA/DHA redox pair balance alters gene expression and protein levels, enhancing stress tolerance (Miret and Müller, 2017). This outcome was also observed in rice, where DHA application triggered systemic resistance against nematodes (Chavan et al., 2022). These findings support our hypothesis that a change in the AsA/DHA redox state is involved in the resistance of the R-PH 16 to *P. citrophthora*.

## 5 Conclusion

**Figure 9:**
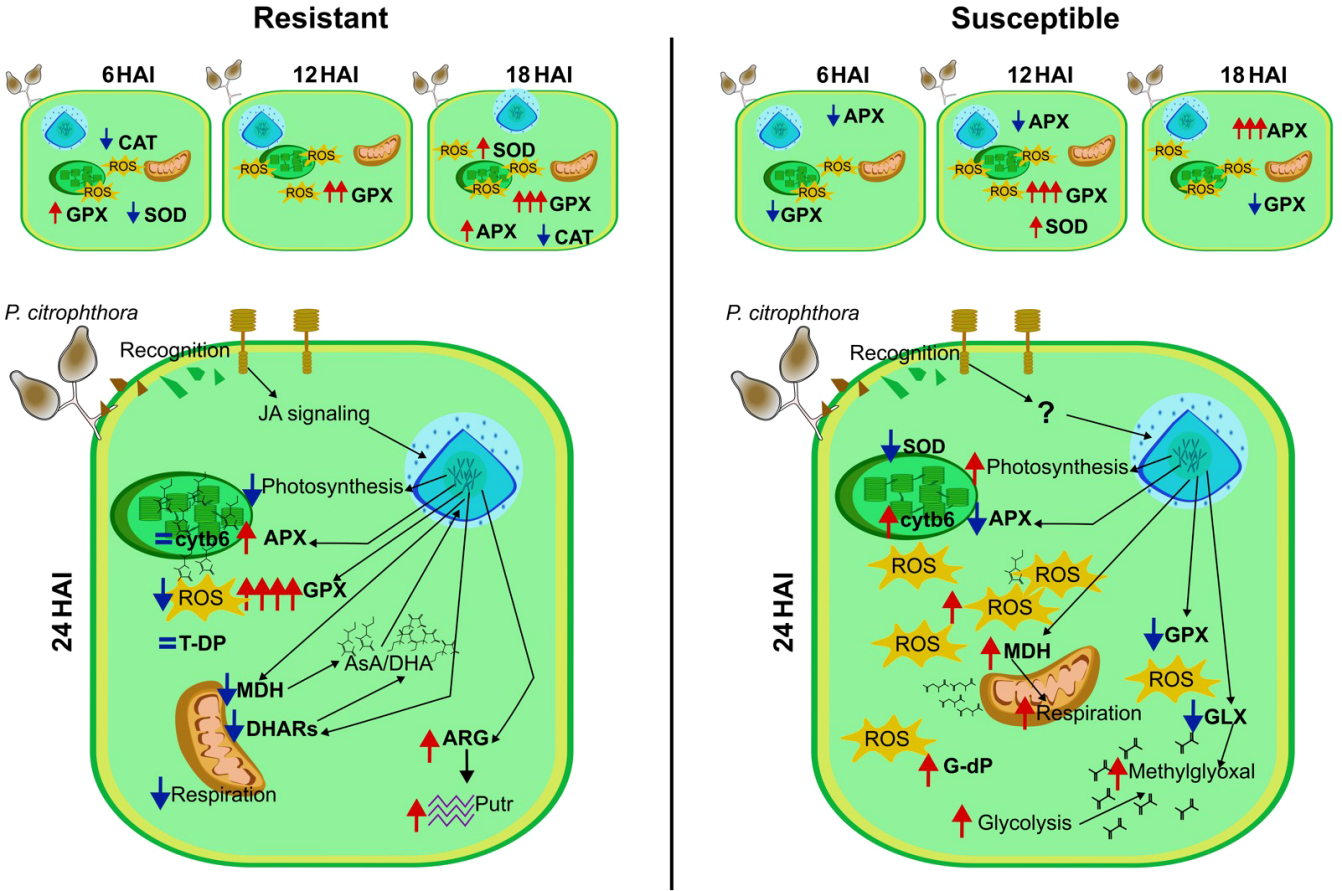
Cellular model of *T. cacao* responses to *P. citrophthora*. The protein and enzyme profiles of the resistant cultivar (PH 16) and the susceptible cultivar (SJ 02) show changes in cell metabolism, differing between the cultivars after inoculation with the oomycete *P. citrophthora*. Red arrows indicate an increase, and blue arrows indicate a decrease in protein accumulation. HAI, hours after inoculation; CAT, catalase enzyme activity; SOD, superoxide dismutase enzyme activity; APX, ascorbate peroxidase enzyme activity; GPX, guaiacol peroxidase enzyme activity; ROS, reactive oxygen species; JA, jasmonic acid; cytb6, cytochrome b6 protein; T-DP, thioredoxin-dependent peroxiredoxin protein; MDH, malate dehydrogenase protein; DHAR, dehydroascorbate reductase protein; AsA, ascorbate; DHA, dehydroascorbate; ARG, arginase protein; Putr, putrescine; G-dP, glutaredoxin-dependent peroxiredoxin protein; GLX, glyoxalase.

After inoculation with *P. citrophthora*, the enzymatic and proteomic profiles were altered in both resistant and susceptible cultivars. Based on the protein abundance profile, we proposed a possible model of these cultivars’ responses to Black pod (**Figure 9**).

The resistant cultivar exhibited a distinct profile of antioxidant enzymes, with GPX being the most active throughout all evaluation periods, which might be crucial for pathogen resistance. Based on the protein profile, we suggest that the R-PH 16 activated the JA signaling pathway, leading to changes in gene expression and, consequently, in protein abundance. In turn, the proteins with altered abundance patterns modified the plant’s metabolism and the AsA/DHA ratio, potentially signaling the activation of other defense-related genes, such as arginine, which induces the accumulation of the polyamine putrescine. This compound could play a significant role in cellular protection. The metabolism of the R-PH 16 changes at 24 HAI, showing evidence of decreased photosynthesis and a shift towards non-assimilatory carbohydrate consumption, which might be related to the induction of defense mechanisms. Proteins constitutively more abundant could be involved in a more efficient response in resistant plants, such as thioredoxin-dependent peroxiredoxin, cytochrome b6, and DHARs.

The S-SJ 02 infected shows few proteins with altered abundance at 24 HAI. The modified proteins indicate an increase in the metabolism of small molecules, such as malate and fructose, as well as an increase in the abundance of proteins involved in photosynthesis, unlike what is observed in the R-PH 16. The decrease of a GLX likely induces an accumulation of the cytotoxic substance methylglyoxal. Additionally, the GPX activity was reduced in this treatment, contrary to what was observed in the R-PH 16. These factors may be related to the susceptibility of the SJ 02 cultivar to *P. citrophthora*.

Taken together, our results demonstrate that the resistant cultivar possibly mobilizes early antioxidant defenses and undergoes metabolic reprogramming to combat infection, while the susceptible cultivar exhibits insufficient responses, leading to cellular damage. These findings provide new insights into cacao-*P. citrophthora* interactions and may provide a foundation for future transcript-level studies aimed at clarifying the gene expression mechanisms underlying these defense responses. Additionally, our findings may support the development of new pre-breeding stages for cacao cultivars.

## Data Availability

The original contributions presented in the datasets supporting this study are included in the article. The row data is available in doi: 10.25345/C54F1MZ0M.

## References

[B1] Adeniyi D. (2019). “ Diversity of Cacao Pathogens and Impact on Yield and Global Production,” in Theobroma Cacao - Deploying Science for Sustainability of Global Cocoa Economy. Ed. Osobase Aikpokpodion P. (London: IntechOpen). doi: 10.5772/intechopen.81993, PMID:

[B2] Alscher R. G. Erturk N. Heath L. S. (2002). Role of superoxide dismutases (SODs) in controlling oxidative stress in plants. J. Exp. Botany. 53, 1331–1341. doi: 10.1093/jexbot/53.372.1331, PMID: 11997379

[B3] Appiah A. A. Flood J. Archer S. A. Bridge P. D. (2004). Molecular analysis of the major *Phytophthora* species on cocoa. Plant Pathology. 53, 209–219. doi: 10.1111/j.0032-0862.2004.00980.x, PMID: 41778641

[B4] Barreto M. A. Rosa J. R. B. F. Holanda I. S. A. Cardoso-Silva C. B. Vildoso C. I. A. Ahnert D. . (2018). QTL mapping and identification of corresponding genomic regions for black pod disease resistance to three Phytophthora species in *Theobroma cacao* L. Euphytica. 214, 188. doi: 10.1007/s10681-018-2273-5, PMID: 41776007

[B5] Baruah I. K. Ali S. S. Shao J. Lary D. Bailey B. A. (2022). Changes in gene expression in leaves of cacao genotypes resistant and susceptible to *phytophthora palmivora* infection. Front. Plant Sci. 12, 780805. doi: 10.3389/fpls.2021.780805, PMID: 35211126 PMC8861199

[B6] Belete T. (2022). A critical review on defense mechanisms of plants against bacterial pathogens: from morphological to molecular levels. J. Plant Pathol. Microbiol. 12, 534. doi: 10.35248/2157-7471.21.12.534

[B7] Bertolde F. Z. Almeida A.-A. F. Corrêa R. X. Gomes F. P. Gaiotto F. A. Baligar V. C. . (2010). Molecular, physiological and morphological analysis of waterlogging tolerance in clonal genotypes of *Theobroma cacao* L. Tree Physiol. 30, 56–67. doi: 10.1093/treephys/tpp101, PMID: 19959598

[B8] Caverzan A. Passaia G. Rosa S. B. Ribeiro C. W. Lazzarotto F. Margis-Pinheiro M. (2012). Plant responses to stresses: Role of ascorbate peroxidase in the antioxidant protection. Genet. Mol. Biol. 35, 1011–1019. doi: 10.1590/S1415-47572012000600016, PMID: 23412747 PMC3571416

[B9] Chavan S. N. De Kesel J. Desmedt W. Degroote E. Singh R. R. Nguyen G. T. . (2022). Dehydroascorbate induces plant resistance in rice against root-knot nematode Meloidogyne graminicola. Mol. Plant Pathology. 23, 1303–1319. doi: 10.1111/mpp.13230, PMID: 35587614 PMC9366072

[B10] Chen Y. Fu Z. Zhang H. Tian R. Yang H. Sun C. . (2020). Cytosolic malate dehydrogenase 4 modulates cellular energetics and storage reserve accumulation in maize endosperm. Plant Biotechnol. J. 18, 2420–2435. doi: 10.1111/pbi.13416, PMID: 32436613 PMC7680550

[B11] Chen H. McCaig B. C. Melotto M. He S. Y. Howe G. A. (2004). Regulation of plant arginase by wounding, jasmonate, and the phytotoxin coronatine. J. Biol. Chem. 279, 45998–46007. doi: 10.1074/jbc.M407151200, PMID: 15322128

[B12] Decloquement J. Ramos-Sobrinho R. Elias S. G. Britto D. S. Puig A. S. Reis A. . (2022). *Phytophthora theobromicola* sp. nov.: A New Species Causing Black Pod Disease on Cacao in Brazil. Front. Microbiol. 12. doi: 10.3389/fmicb.2021.537399, PMID: 33815301 PMC8015942

[B13] Dixon D. P. Davis B. G. Edwards R. (2002). Functional divergence in the glutathione transferase superfamily in plants. J. Biol. Chem. 277, 30859–30869. doi: 10.1074/jbc.M202919200, PMID: 12077129

[B14] Freitas L. S. Silva G. S. Santos I. C. D. Ferreira A. C. R. Santos L. E. S. Umaharan P. . (2025). Elite cacao clonal cultivars with diverse genetic structure, high potential of production, and good organoleptic quality are helping to rebuild the cocoa industry in Brazil. Int. J. Mol. Sci. 26, 3386. doi: 10.3390/ijms26073386, PMID: 40244280 PMC11989740

[B15] Ge S. X. Jung D. Yao R. (2019). ShinyGO: a graphical gene-set enrichment tool for animals and plants. Bioinformatics. 36, 2628–2629. doi: 10.1093/bioinformatics/btz931, PMID: 31882993 PMC7178415

[B16] Giannopolitis C. N. Ries S. K. (1977). Superoxide dismutases. Plant Physiol. 59, 309–314. doi: 10.1104/pp.59.2.309, PMID: 16659839 PMC542387

[B17] González-Hernández A. I. Scalschi L. Vicedo B. Marcos-Barbero E. L. Morcuende R. Camañes G. (2022). Putrescine: A key metabolite involved in plant development, tolerance and resistance responses to stress. Int. J. Mol. Sci. 23, 2971. doi: 10.3390/ijms23062971, PMID: 35328394 PMC8955586

[B18] Guest D. (2007). Black pod: diverse pathogens with a global impact on cocoa yield. Phytopathology. 97, 1650–1653. doi: 10.1094/PHYTO-97-12-1650, PMID: 18943728

[B19] Havir E. A. McHale N. A. (1987). Biochemical and developmental characterization of multiple forms of catalase in tobacco leaves. Plant Physiol. 84, 450–455. doi: 10.1104/pp.84.2.450, PMID: 16665461 PMC1056601

[B20] Hoque T. S. Hossain M. A. Mostofa M. G. Burritt D. J. Fujita M. Tran L.-S. P. (2016). Methylglyoxal: an emerging signaling molecule in plant abiotic stress responses and tolerance. Front. Plant Sci. 7. doi: 10.3389/fpls.2016.01341, PMID: 27679640 PMC5020096

[B21] Hossain M. A. Bhattacharjee S. Armin S.-M. Qian P. Xin W. Li H.-Y. . (2015). Hydrogen peroxide priming modulates abiotic oxidative stress tolerance: insights from ROS detoxification and scavenging. Front. Plant Sci. 6. doi: 10.3389/fpls.2015.00420, PMID: 26136756 PMC4468828

[B22] Jung C. Lyou S. H. Yeu S. Kim M. A. Rhee S. Kim M. . (2007). Microarray-based screening of jasmonate-responsive genes in Arabidopsis thaliana. Plant Cell Rep. 26, 1053–1063. doi: 10.1007/s00299-007-0311-1, PMID: 17297615

[B23] Kalisz A. Sekara A. Pokluda R. Jezdinský A. Neugebauerová J. Slezák K. A. . (2019). Sequential response of sage antioxidant metabolism to chilling treatment. Molecules. 24, 4087. doi: 10.3390/molecules24224087, PMID: 31726737 PMC6891540

[B24] Laemmli U. K. (1970). Cleavage of structural proteins during the assembly of the head of bacteriophage T4. Nature. 227, 680–685. doi: 10.1038/227680a0, PMID: 5432063

[B25] Lopez U. V. Paim M. Luz E. D. M. N. Silva S. D. V. M. Gramacho K. P. Pires J. L. . (2006). “ Resistance of cacao farmer selections to *Phytophthora citrophthora* in Brazil.” 15th International Cocoa Research Conference, 2007, San José, Costa Rica. Proceedings. (Nogéria: COPAL, Academy Press Plc), 1, 21–25.

[B26] Luz E. D. M. N. Mitchell D. J. . (1994). Effects of Inoculum Forms and Densities on Cacao Root Infection by Phytophthora spp. Agrotrópica 6, 41–5l.

[B27] Luz E. D. M. N. Silva S. D. V. M. Bezerra J. L. Souza J. T. Santos A. F. D. (2008). Glossário Ilustrado de Phytophthora: Técnicas Especiais para o Estudo de Oomicetos (Itabuna, BA: Fapesb).

[B28] Maere S. Heymans K. Kuiper M. (2015). BiNGO: a Cytoscape plugin to assess overrepresentation of Gene Ontology categories in biological networks. Bioinformatics. 21, 3448–3449. doi: 10.1093/bioinformatics/bti551, PMID: 15972284

[B29] Marelli J.-P. Guest D. I. Bailey B. A. Evans H. C. Brown J. K. Junaid M. . (2019). Chocolate Under Threat from Old and New Cacao Diseases. Phytopathology 109, 1331–1343. doi: 10.1094/PHYTO-12-18-0477-RVW, PMID: 31115251

[B30] Mchau G. R. A. Coffey M. D. (1994). An integrated study of morphological and isozyme patterns found within a worldwide collection of *Phytophthora citrophthora* and a redescription of the species. Mycological Res. 98, 1291–1299. doi: 10.1016/S0953-7562(09):80301-8, PMID: 41737640

[B31] McMahon P. Purwantara A. (2004). “ *Phytophthora* on cocoa,” in Diversity and Management of Phytophthora in Southeast Asia. eds. Drenth A. Guest D. I. (Canberra: Australian Centre for International Agricultural Research), 104–115.

[B32] Miret J. A. Müller M. (2017). “ AsA/DHA Redox Pair Influencing Plant Growth and Stress Tolerance,” in Ascorbic Acid in Plant Growth, Development and Stress Tolerance. (Cham: Springer International Publishing), 297–319. doi: 10.1007/978-3-319-74057-7_12, PMID:

[B33] Mora-Ocampo I. Y. (2020). *Respostas de defesa no xilema e uma nova proteína intrinsecamente desestruturada participam na resistência do cacaueiro à murcha de Ceratocystis*. (Doctoral Thesis). (Ilhéus, BA: Universidade Estadual de Santa Cruz).

[B34] Mora-Ocampo I. Y. Pirovani C. P. Luz E. D. M. N. Rêgo A. P. B. Silva E. M. A. Rhodes-Valbuena M. . (2022). *Ceratocystis cacaofunesta* differentially modulates the proteome in xylem-enriched tissue of cocoa genotypes with contrasting resistance to Ceratocystis wilt. Planta. 254, 94. doi: 10.1007/s00425-021-03747-5, PMID: 34642817

[B35] Moreno-Chacón A. L. Camperos-Reyes J. E. Diaz-granados R. A. A. Romero H. M. (2013). Biochemical and physiological responses of oil palm to bud rot caused by Phytophthora palmivora. Plant Physiol. Biochem. 70, 246–251. doi: 10.1016/j.plaphy.2013.05.026, PMID: 23796724

[B36] Munekage Y. Takeda S. Endo T. Jahns P. Hashimoto T. Shikanai T. (2002). Cytochrome b6f mutation specifically affects thermal dissipation of absorbed light energy in Arabidopsis. Plant J. 28, 351–359. doi: 10.1046/j.1365-313X.2001.01178.x, PMID: 11722777

[B37] Nakano Y. Asada K. (1987). Purification of ascorbate peroxidase in spinach chloroplasts; its inactivation in ascorbate-depleted medium and reactivation by monodehydroascorbate radical. Plant Cell Physiol. 28, 131–140. doi: 10.1093/oxfordjournals.pcp.a077268, PMID: 41777722

[B38] Nepusz G. C. A. T. Csárdi G. (2006). The igraph software package for complex network research. Complex Systems. 1695, 1–9.

[B39] Neuhoff V. Arold N. Taube D. Ehrhardt W. (1988). Improved staining of proteins in polyacrylamide gels including isoelectric focusing gels with clear background at nanogram sensitivity using Coomassie Brilliant Blue G-250 and R-250. Electrophoresis. 9, 255–262. doi: 10.1002/elps.1150090603, PMID: 2466658

[B40] Nunes-Nesi A. Carrari F. Lytovchenko A. Smith A. M. O. Ehlers Loureiro M. Ratcliffe R. G. . (2005). Enhanced photosynthetic performance and growth as a consequence of decreasing mitochondrial malate dehydrogenase activity in transgenic tomato plants. Plant Physiol. 137, 611–622. doi: 10.1104/pp.104.055566, PMID: 15665243 PMC1065362

[B41] Perrine-Walker F. (2020). Phytophthora palmivora–cocoa interaction. J. Fungi. 6, 167. doi: 10.3390/jof6030167, PMID: 32916858 PMC7558484

[B42] Pirovani C. P. Carvalho H. A. S. Machado R. C. R. Gomes D. S. Alvim F. C. Pomella A. W. V. . (2008). Protein extraction for proteome analysis from cacao leaves and meristems, organs infected by *Moniliophthora perniciosa*, the causal agent of the witches’ broom disease. Electrophoresis. 29, 2391–2401. doi: 10.1002/elps.200700743, PMID: 18435495

[B43] R Core Team (2022). R: A Language and Environment for Statistical Computing (4.0.3) (Vienna: R Foundation for Statistical Computing (Org). Available online at: https://www.r-project.org/ (Accessed October 31, 2022).

[B44] Rêgo A. P. B. Mora-Ocampo I. Y. Corrêa R. X. (2023). Interactions of different species of phytophthora with cacao induce genetic, biochemical, and morphological plant alterations. Microorganisms. 11, 1172. doi: 10.3390/microorganisms11051172, PMID: 37317146 PMC10221226

[B45] Rego A. P. B. Mora-Ocampo I. Y. Pirovani C. P. Luz E. D. M. N. Corrêa R. X. (2022). Protein Level Defense Responses of Theobroma cacao Interaction with Phytophthora palmivora. Front. Agron. 4. doi: 10.3389/fagro.2022.836360, PMID: 41778173

[B46] Rehem B. C. Almeida A. A. F. Santos I. C. Gomes F. P. Pirovani C. P. Mangabeira P. A. O. . (2012). Photosynthesis, chloroplast ultrastructure, chemical composition and oxidative stress in Theobroma cacao hybrids with the lethal gene Luteus-Pa mutant. Photosynthetica. 49, 127–139. doi: 10.1007/978-3-319-02294-9-72, PMID: 41776007

[B47] Rouhier N. Gelhaye E. Jacquot J. P. (2002). Glutaredoxin-dependent peroxiredoxin from poplar. J. Biol. Chem. 277, 13609–13614. doi: 10.1074/jbc.M111489200, PMID: 11832487

[B48] Saha J. Brauer E. K. Sengupta A. Popescu S. C. Gupta K. Gupta B. . (2015). Polyamines as redox homeostasis regulators during salt stress in plants. Front. Environ. Sci. 3. doi: 10.3389/fenvs.2015.00021, PMID: 41778173

[B49] Santos E. S. L. Cerqueira-Silva C. B. M. Mori G. M. Ahnert D. Mello D. L. N. Pires J. L. . (2015). Genetic structure and molecular diversity of cacao plants established as local varieties for more than two centuries: the genetic history of cacao plantations in Bahia, Brazil. PloS One 10, e0145276. doi: 10.1371/journal.pone.0145276, PMID: 26675449 PMC4682715

[B50] Santos E. C. Pirovani C. P. Correa S. C. Micheli F. Gramacho K. P. (2020). The pathogen *Moniliophthora perniciosa* promotes differential proteomic modulation of cacao genotypes with contrasting resistance to witches´ broom disease. BMC Plant Biol. 20, 1. doi: 10.1186/s12870-019-2170-7, PMID: 31898482 PMC6941324

[B51] Scharte J. Schön H. Weis E. (2005). Photosynthesis and carbohydrate metabolism in tobacco leaves during an incompatible interaction with *Phytophthora nicotianae*. Plant Cell Environment. 28, 1421–1435. doi: 10.1111/j.1365-3040.2005.01380.x, PMID: 41778641

[B52] Shaikhali J. Heiber I. Seidel T. Ströher E. Hiltscher H. Birkmann S. . (2008). The redox-sensitive transcription factor Rap2.4a controls the nuclear expression of 2-Cys peroxiredoxin A and other chloroplast antioxidant enzymes. BMC Plant Biol. 8, 48. doi: 10.1186/1471-2229-8-48, PMID: 18439303 PMC2386467

[B53] Shannon P. (2003). Cytoscape: A software environment for integrated models of biomolecular interaction networks. Genome Res. 13, 2498–2504. doi: 10.1101/gr.1239303, PMID: 14597658 PMC403769

[B54] Smith R. E. Smith H. E. . (1906). A new fungus of economic importance. Bot. Gaz. 42, 215–221. Available online at: https://hdl.handle.net/2027/uc1.c027367097.

[B55] Sodré G. A. Marrocos P. C. L. (2009). Manual da produção vegetativa de mudas de cacaueiro: produção de mudas enxertadas em viveiro. (Ilhéus: Editus). Available online at: https://www.uesc.br/editora/livrosdigitais2015/manual_da_producao.pdf.

[B56] Surujdeo-Maharaj S. Sreenivasan T. N. Motilal L. A. Umaharan P. (2016). “ Black pod and other phytophthora induced diseases of cacao: history, biology, and control,” in Cacao diseases (Cham: Springer International Publishing), 213–266. doi: 10.1007/978-3-319-24789-2_7, PMID:

[B57] Tesfaye M. Temple S. J. Allan D. L. Vance C. P. Samac D. A. (2001). Overexpression of malate dehydrogenase in transgenic alfalfa enhances organic acid synthesis and confers tolerance to aluminum. Plant Physiol. 127, 1836–1844. doi: 10.1104/pp.010376 11743127 PMC133587

[B58] Tomaz T. Bagard M. Pracharoenwattana I. Lindén P. Lee C. P. Carroll A. J. . (2010). Mitochondrial malate dehydrogenase lowers leaf respiration and alters photorespiration and plant growth in arabidopsis. Plant Physiol. 154, 1143–1157. doi: 10.1104/pp.110.161612, PMID: 20876337 PMC2971595

[B59] Villén J. Gygi S. P. (2008). The SCX/IMAC enrichment approach for global phosphorylation analysis by mass spectrometry. Nat. Protoc. 3, 1630–1638. doi: 10.1038/nprot.2008.150, PMID: 18833199 PMC2728452

[B60] Wang Q.-F. Zhao Y. Yi Q. Li K.-Z. Yu Y. X. Chen L. M. . (2010). Overexpression of malate dehydrogenase in transgenic tobacco leaves: enhanced malate synthesis and augmented Al-resistance. Acta Physiol. Plant 32, 1209–1220. doi: 10.1007/s11738-010-0522-x, PMID: 41776007

[B61] Winters N. P. Wafula E. K. Knollenberg B. J. Hämälä T. Timilsena P. R. Perryman M. . (2024). A combination of conserved and diverged responses underlies *Theobroma cacao*’s defense response to *Phytophthora palmivora*. BMC Biol. 22, 38. doi: 10.1186/s12915-024-01831-2, PMID: 38360697 PMC10870529

[B62] Zhang Y. Fernie A. R. (2018). On the role of the tricarboxylic acid cycle in plant productivity. J. Integr. Plant Biol. 60, 1199–1216. doi: 10.1111/jipb.12690, PMID: 29917310

